# Supplementary material for: Halogenated Pyrrolopyrimidines with Low MIC on Staphylococcus aureus and Synergistic Effects with an Antimicrobial Peptide
Source: Antibiotics (Basel). 2022 Jul 22;11(8):984. doi: 10.3390/antibiotics11080984 (PMC9330635; doi:10.3390/antibiotics11080984)
Supplement: Supplementary file 1 [file antibiotics-11-00984-s001.zip › antibiotics-1826880-supplementary.pdf]

Supporting information

Halogenated pyrrolopyrimidines with low MIC on *Staphylococcus aureus* and synergistic effects with an antimicrobial peptide

Cecilie Elisabeth Olsen[1], Fredrik Heen Blindheim[1], Caroline Krogh Søgaard[2], Lisa Marie Røst[2], Amanda Holstad Singleton[2], Olaug Elisabeth Torheim Bergum[2], Per Bruheim[3], Marit Otterlei[2], Eirik Sundby[4], Bård Helge Hoff[2,*]

Contents

Synthesis of building blocks 2

NMR spectroscopy 12

Comparison of TMPK folding 32

References 32

Synthesis of building blocks

4-Chloro-7-((2-(trimethylsilyl)ethoxy)methyl)-7*H*-pyrrolo[2,3-*d*]pyrimidine[1]

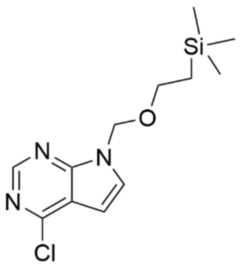

The following procedure was adapted from Reiersølmoen *et al.*[2] 4-Chloro-7*H*-pyrrolo[2,3-*d*]pyrimidine (3.06 g, 19.9 mmol) was dissolved in dry DMF (20 mL) and stirred at 0 °C under and N_2 atmosphere. To the mixture was added NaH (578 mg, 24.1 mmol) dissolved in dry DMF (50 mL) dropwise over 10 minutes. The solution was left to clear for 30 minutes before SEM-Cl (4.58 mL, 25.9 mmol) was added dropwise over 30 minutes. The solution was stirred for an additional 2.5 hours, and the reaction was subsequently cooled to 0 °C and quenched with H_2O (500 mL). The resulting slurry was extracted with EtOAc (4 ×100 mL). The combined organic layers were dried over anhydrous Na_2SO_4, filtered and concentrated in vacuo. The crude product was then purified twice by flash chromatography (silica-gel, EtOAc/*n*-pentane, 0:1 to 1:2), resulting in 4.06 g (14.3 mmol, 72%) of the desired product as a waxy solid. TLC (EtOAc/*n*-pentane 1:8) R_f = 0.20; ^{1}H NMR (400 MHz, DMSO-d_6) δ: 8.68 (s, 1H), 7.87 (d, *J* = 3.7 Hz, 1H), 6.71 (d, *J* = 3.7 Hz, 1H), 5.64 (s, 2H), 3.61 – 3.43 (m, 2H), 0.93 – 0.73 (m, 2H), -0.12 (s, 9H). The ^{1}H-NMR spectrum was in accordance with the literature [1].

4-Chloro-6-iodo-7-((2-(trimethylsilyl)ethoxy)methyl)-7*H*-pyrrolo[2,3-*d*]pyrimidine (22)[3]

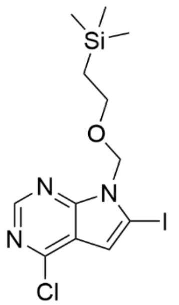

The procedure is an adaptation from Reiersølmoen *et al.*[2] A solution of 4-chloro-6-iodo-7-((2-(trimethylsilyl)ethoxy)methyl)-7*H*-pyrrolo[2,3-*d*]pyrimi-dine (3.51 g, 12.4 mmol) in dry THF (30 mL) was cooled to -78 °C and stirred under an N_2 atmosphere. To this was added a solution of LDA in THF (2.0 M, 9.28 mL) dropwise over 30 minutes. The solution was stirred for a further 30 minutes, before I_2 (3.47 g, 13.6 mmol) dissolved in THF (10 mL) was added dropwise over 30 minutes. The solution was then stirred for further 30 minutes before quenching with NH_4Cl (6 M, 3.1 mL) and warming to ambient temperature. Sodium thiosulphate solution (10%, 27 mL) and CH_2Cl_2 (50 mL) was then added before the phases were separated. The aqueous phase was then extracted with CH_2Cl_2 (4 × 50 mL), and the combined organic layers dried over anhydrous Na_2SO_4, filtered and concentrated *in vacuo*. The resulting crude material was purified twice by gradient flash chromatography (silica, EtOAc/n-pentane, 0:1 to 1:10) resulting in 4.41 g (10.8 mmol, 87%) of the desired product as brown crystals. TLC (EtOAc/*n*-pentane 1:10) R_f = 0.13; ^{1}H NMR (400 MHz, DMSO-d_6) δ: 8.64 (s, 1H), 7.13 (s, 1H), 5.62 (s, 2H), 3.58 – 3.49 (m, 2H), 0.87 – 0.79 (m, 2H), -0.10 (s, 9H). The ^{1}H-NMR spectrum was in accordance with the literature.[3]

4-Chloro-6-(4-methoxyphenyl)-7-((2-(trimethylsilyl)ethoxy)methyl)-7*H*-pyrrolo[2,3-*d*]-pyrimidine (23)[4]

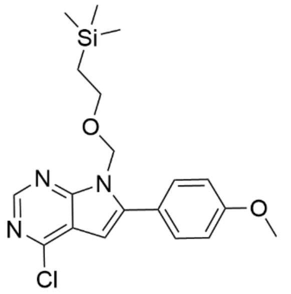

4-Chloro-6-(4-methoxyphenyl)-7-((2-(ethylsilyl)ethoxy)methyl)-7*H*-pyrrolo[2,3-*d*]pyrimidine (251 mg, 0.613 mmol), (4-methoxyphenyl)boronic acid (102.9 mg, 0.677 mmol), K_2CO_3 (253 mg, 1.83 mmol) and Pd(dppf)Cl_2 (22.4 mg, 30.6 µmol) was added to a Schlenk tube and flushed with N_2. Degassed H_2O (2.5 mL) and 1,4-dioxane (2.5 mL) was added, and the reaction was stirred at 80 °C for 10 minutes. After cooling to ambient temperature, H_2O (25 mL) and CH_2Cl_2 (25 mL) was added, and the phases separated. The aqueous phase was extracted with CH_2Cl_2 (3 × 25 mL) and the combined organic layers washed with brine (25 mL). They were then dried over Na_2SO_4, filtered and concentrated *in vacuo*. This crude was purified by gradient flash chromatography (*i*-PrOH/CH_2Cl_2, 0:100 to 8:92), resulting in 160 mg (0.409 mmol, 67%) of the desired product as a brown oil. TLC (iPrOH/CH_2Cl_2 2:98) R_f = 0.34; ^{1}H NMR (400 MHz, $CDCl_3$) δ: 8.65 (s, 1H), 7.76 – 7.69 (m, 2H), 7.06 – 7.00 (m, 2H), 6.63 (s, 1H), 5.61 (s, 2H), 3.89 (s, 3H), 3.78 – 3.71 (m, 2H), 1.01 – 0.95 (m, 2H), -0.02 (s, 9H). The ^{1}H-NMR spectrum was in accordance with the literature.[4]

(4-(4-Chloro-7-((2-(trimethylsilyl)ethoxy)methyl)-7*H*-pyrrolo[2,3-*d*]pyrimidin-6-yl)phenol (24)[3]

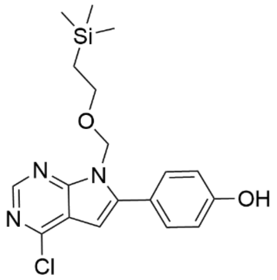

A mixture of 4-chloro-6-iodo-7-((2-(trimethylsilyl)ethoxy)methyl) -7*H*-pyrrolo[2,3-*d*]pyrimidine (2.05 g, 5.01 mmol), (4-hydroxy- phenyl)boronic acid (814 mg, 5.90 mmol), K_2CO_3 (1.39 g, 10.1 mmol) and Pd_2dba_3 (113 mg, 0.123 mmol) was flushed with N_2 three times. Degassed 1,4 dioxane (9 mL) and H_2O (3 mL) were then added, and the mixture stirred at 60 °C for 2.5 hours. Additional degassed 1,4 dioxane (9 mL) and H_2O (3 mL) were then added, and the solution stirred for a further 2.5 hours. After cooling to ambient temperature, the solvent was removed *in vacuo*, and the resulting solid suspended in H_2O (100 mL). The suspension was extracted with EtOAc (4 ×100 mL) and the combined organic layers washed with brine (100 mL), dried over anhydrous Na_2SO_4, filtered and concentrated *in vacuo*. The resulting crude was immobilized on Celite and purified by gradient flash chromatography (EtOAc/*n*-pentane/AcOH, 10:100:1 to 25:100:1) yielding 1.34 g (3.56 mmol, 71%) of the desired product as a light brown solid. TLC (AcOH/EtOAc/*n*-pentane 1:80:10) R_f = 0.22; ^{1}H NMR (400 MHz, DMSO-d_6) δ: 9.94 (s, 1H), 8.68 (s, 1H), 7.72 – 7.61 (m, 2H), 7.01 – 6.89 (m, 2H), 6.74 (s, 1H), 5.60 (s, 2H), 3.68 – 3.57 (m, 2H), 0.89 – 0.80 (m, 2H), -0.10 (s, 9H). The ^{1}H-NMR spectrum was in accordance with the literature.[3]

(*S*)-*N*-(1-(4-Bromophenyl)ethyl)-6-iodo-7-((2-(trimethylsilyl)ethoxy)methyl)-7*H*-pyrrolo[2,3-*d*]pyrimidin-4-amine ((*S*)-34)

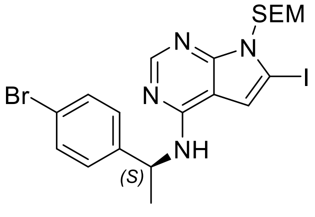

4-Chloro-6-iodo-7-((2-(trimethylsilyl)ethoxy)methyl)-7*H*-pyrrol-o[2,3-*d*]pyrimidine (**22**) (503 mg, 1.23 mmol) was treated as described in General Procedure A. The crude material was purified by gradient flash chromatography (silica-gel, Et_2O/CH_2Cl_2, 0:100 to 1:3) to afford 541 mg (0.944 mmol, 77%) as an off-white waxy solid; TLC (Et_2O/CH_2Cl_2, 1:3): R_F = 0.42; $[\alpha]_D^{20}$ = +61.0 (1.00, $CHCl_3$); ^{1}H NMR (600 MHz, DMSO-d_6) δ: 8.03 (s, 1H), 7.90 (d, *J* = 7.9 Hz, 1H), 7.49-7.47 (m, 2H), 7.34-7.33 (m, 2H), 7.11 (s, 1H), 5.47-5.39 (m, 3H),

3.50-3.47 (m, 2H), 1.49 (d, *J* = 7.0 Hz, 3H), 0.81-0.78 (m, 2H), -0.10 (s, 9H); ^{13}C NMR (150 MHz, DMSO-*d*$_6$) δ: 153.7, 151.9, 151.4, 144.7, 131.1 (2C), 128.3 (2C), 119.4, 110.1, 104.7, 79.8, 72.5, 65.4, 48.4, 22.6, 17.1, -1.4 (3C); IR (neat, cm^{-1}): 3281, 2951, 2893, 1597, 1563, 1504, 1488, 1454, 1378, 1339, 1297, 1248, 1217, 1077, 1010, 859, 835, 749; HRMS (ASAP-TOF, *m/z*): calcd. for $C_{20}H_{27}N_4OSi^{79}BrI$, 573.0182 [M+H]$^+$, found 573.1087.

(*R*)-4-(4-((1-(4-Bromophenyl)ethyl)amino)-7-((2-(trimethylsilyl)ethoxy)methyl)-7*H*-pyrrolo[2,3-*d*]pyrimidin-6-yl)phenol (34)

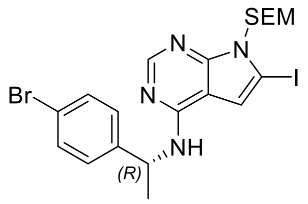

Compound **34** was made as described in General Procedure A starting with 4-chloro-6-iodo-7-((2-(trimethylsilyl)ethoxy)methyl)-7*H*-pyrrolo[2,3-*d*]pyrimidine (**22**) (1.17 g, 2.87 mmol) and (*R*)-1-(4-bromophenyl)ethan-1-amine (1.34 mL, 9.31 mmol). The product was purified by gradient flash chromatography (silica-gel, Et_2O/CH_2Cl_2, 0:100 to 1:3) to afford 541 mg (0.944 mmol, 77%) as an off-white waxy solid; TLC (Et_2O/CH_2Cl_2, 1:3): R_F = 0.42. This gave 1.49 g (2.60 mmol, 91%) as an off-white waxy solid; $[\alpha]_D^{20}$ = – 61.2 (1.00, $CHCl_3$). ^{1}H NMR spectroscopy corresponded with that reported for the (*S*)-enantiomer.

4-Chloro-6-(4-methoxyphenyl)thieno[2,3-*d*]pyrimidine (44)[5]

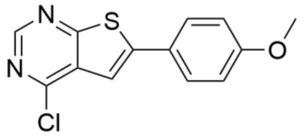

6-Bromo-4-chlorothieno[2,3-*d*]pyrimidine[6] (200 mg, 0.801 mmol), phenylboronic acid (150 mg, 0.987 mmol), K_2CO_3 (350 mg, 2.53 mmol), $Pd_2(dba)_3$ (41 mg, 44.7 µmol) and degassed 1,4-dioxane (4 mL) were mixed and stirred at 130 °C for 4.5 h under an N_2 atmosphere. The solvent was evaporated under reduced pressure. The residue was suspended in water (50 mL), extracted with Et_2O (3 × 50 mL), dried over $MgSO_4$ and filtered. The solvent was evaporated under reduced pressure. The crude product was purified by flash chromatography (silica, *n*-pentane/acetone, 20:1, R_f = 0.20) to give 163 mg (0.590 mmol, 73%) of the product. ^{1}H NMR (400 MHz, $CDCl_3$) δ: 8.79 (s, 1H), 7.68 (d, *J* = 8.8 Hz, 1H), 7.47 (s, 1H), 7.00 (d, *J* = 8.8 Hz, 1H), 3.88 (s, 1H).
The ^{1}H NMR spectrum in $CDCl_3$ was similar to a previously published DMSO-*d*$_6$ spectrum[5].

Synthesis of SEM protected 6-aryl-7H-pyrrolo[2,3-d]pyrimidin-4-amines

***(R)*-4-(4-((1-Phenylethyl)amino)-7-((2-(trimethylsilyl)ethoxy)methyl)-7*H*-pyrrolo[2,3-*d*]pyrimidin-6-yl)phenol (25)**

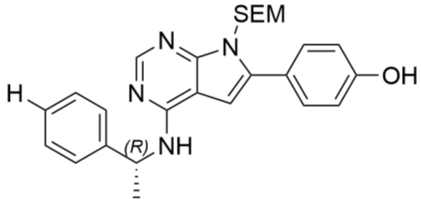

Compound **25** was prepared as described in General Procedure A starting with **24** (139 mg, 0.370 mmol) and *(R)*-1-phenylethan-1-amine (150 µL, 1.18 mmol). The crude product was purified by dissolution in CH_2Cl_2 and purification by two rounds of flash chromatography (first: C18 silica, $MeCN/H_2O$ cont. 0.1% NEt_3, 3:1, then: silica-gel: EtOAc/*n*-pentane, 1:4 to 1:2), resulting in 106 mg (0.230 mmol, 62%) of the desired product as a light brown waxy solid, mp. 83.2-87.1 °C; TLC (EtOAc/*n*-pentane 1:2) R_f = 0.23; ^{1}H NMR (600 MHz, DMSO-*d*$_6$) δ: 9.71 (s, 1H), 8.11 (s, 1H), 7.84 (d, *J* = 8.2 Hz, 1H), 7.55 – 7.48 (m, 2H), 7.45 - 7.39 (m, 2H), 7.34 – 7.27 (m, 2H), 7.22 – 7.16 (m, 1H), 6.90 – 6.84 (m, 2H), 6.78 (s, 1H), 5.50 (p, *J* = 7.2 Hz, 1H), 5.44 (s, 2H), 3.64 - 3.55 (m, 2H), 1.53 (d, *J* = 7.1 Hz, 3H), 0.87 – 0.78 (m, 2H), -0.09 (s, 9H); ^{13}C NMR (151 MHz, DMSO-*d*$_6$) δ: 157.6, 154.8, 151.5, 151.4,

145.4, 137.1, 129.9 (2C), 128.2 (2C), 126.4, 126.0 (2C), 122.4, 115.6 (2C), 102.6, 98.2, 70.1, 65.6, 48.7, 22.8, 17.3, -1.4 (3C); IR (neat, cm^{-1}) : 2951, 1600, 1496, 1470, 1246, 1216, 1074, 858, 699; HRMS (TOF ES+, *m/z*): calcd. for $C_{26}H_{33}N_4O_2Si$ $[M+H]^+$: 461.2373, found: 461.2370.

(*R*)-4-(4-((1-(p-Tolyl)ethyl)amino)-7-((2-(trimethylsilyl)ethoxy)methyl)-7*H*-pyrrolo[2,3-*d*]pyrimidin-6-yl)phenol (26)

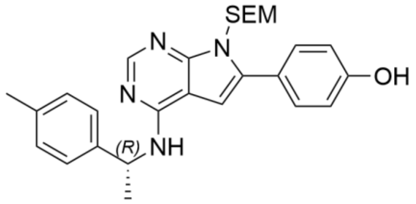

Compound **26** was prepared as described in General Procedure A starting from **24** (202 mg, 0.536 mmol) and *(R)*-1-(*p*-tolyl)ethan-1-amine (220 µL, 1.50 mmol). The crude product was purified by gradient flash chromatography (silica-gel, EtOAc/*n*-pentane, 0:1 to 1:2) resulting in 197 mg (0.415 mmol, 77%) of the desired product as a light brown solid, mp. 88.2-90.3 °C. TLC (EtOAc/*n*-pentane 1:2) R_f = 0.30; ^{1}H NMR (600 MHz, DMSO-*d6*) δ: 9.71 (s, 1H), 8.11 (s, 1H), 7.78 (d, *J* = 8.2 Hz, 1H), 7.54 – 7.48 (m, 2H), 7.32 – 7.26 (m, 2H), 7.13 – 7.07 (m, 2H), 6.89 – 6.85 (m, 2H), 6.77 (s, 1H), 5.49 – 5.42 (m, 3H), 3.64 – 3.52 (m, 2H), 2.25 (s, 3H), 1.50 (d, *J* = 7.0 Hz, 3H), 0.88 – 0.78 (m, 2H), -0.09 (s, 9H); ^{13}C NMR (151 MHz, DMSO-*d6*) δ: 157.5, 154.8, 151.5 (2C), 142.4, 137.0, 135.4, 129.9 (2C), 128.7 (2C), 125.9 (2C), 122.4, 115.5 (2C), 102.6, 98.2, 70.1, 65.6, 48.4, 22.8, 20.6, 17.3, -1.4 (3C); IR (neat, cm^{-1}): 2951, 1600, 1497, 1470, 1342, 1246, 1218, 1074, 833, 764, 736, 601; HRMS (TOF ES+, *m/z*): calcd. for $C_{27}H_{35}N_4O_2Si$ $[M+H]^+$: 475.2529, found: 475.2531.

***(R)*-4-(4-((1-(4-Chlorophenyl)ethyl)amino)-7-((2-(trimethylsilyl)ethoxy)methyl)-7*H*-pyrrolo [2,3-*d*]pyrimidin-6-yl)phenol (27)**

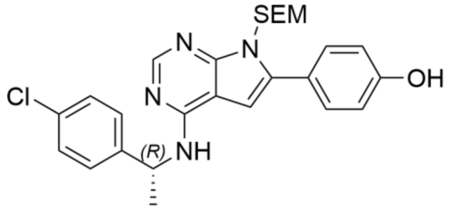

Compound **27** was prepared as described in General Procedure A, starting from **24** (101 mg, 0.256 mmol) and (*R*)-1-(4-chlorophenyl)ethan-1-amine (115 µL, 0.823 mmol).The crude product was purified by dissolution in CH_2Cl_2 and purification by two rounds of flash chromatography (first: silica-gel: MeOH/CH_2Cl_2, 0:100 to 5:95, then: C18-silica, MeCN/H_2O cont. 0.1% NEt_3, 4:1). This gave 65 mg (0.132 mmol, 49%) of the desired product as a brown solid, mp. 91.0-92.7 °C; TLC (MeOH/CH_2Cl_2 3:97) R_f = 0.31; ^{1}H NMR (600 MHz, DMSO-*d6*) δ: 9.72 (s, 1H), 8.11 (s, 1H), 7.87 (d, *J* = 8.0 Hz, 1H), 7.55 – 7.48 (m, 2H), 7.45 – 7.41 (m, 2H), 7.38 – 7.34 (m, 2H), 6.89 – 6.85 (m, 2H), 6.76 (s, 1H), 5.50 – 5.41 (m, 3H), 3.64 – 3.51 (m, 2H), 1.51 (d, *J* = 7.0 Hz, 3H), 0.88 – 0.77 (m, 2H), -0.10 (s, 9H); ^{13}C NMR (151 MHz, DMSO-*d6*) δ: 157.6, 154.7, 151.4 (2C), 144.5, 137.2, 130.9, 129.9 (2C), 128.1 (2C), 127.9 (2C), 122.4, 115.6 (2C), 102.7, 98.1, 70.1, 65.6, 48.3, 22.7, 17.3, -1.4 (3C); IR (neat, cm^{-1}): 2951, 1600, 1493, 1470, 1342, 1075, 858; HRMS (TOF ES+, *m/z*): calcd. for $C_{26}H_{32}N_4O_2Si^{35}Cl$ $[M+H]^+$: 495.1983, found: 495.1985.

(*R*)-4-(4-((1-(4-Bromophenyl)ethyl)amino)-7-((2-(trimethylsilyl)ethoxy)methyl)-7*H*-pyrrolo[2,3-*d*]pyrimidin-6-yl)phenol (28)

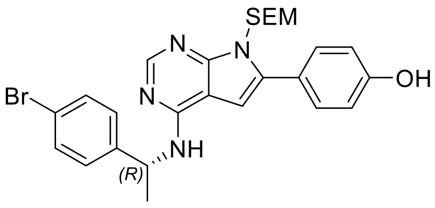

Compound **28** was prepared as described in General Procedure A from **24** (203 mg, 0.540 mmol) and (*R*)-1-(4-bromophenyl)ethan-1-amine (0.235 mL, 1.63 mmol). The crude product was immobilized on Celite and purified by gradient flash chromatography (silica, EtOAc/*n*-pentane 0:1 to 1:1). This gave 203 mg (0.376 mmol, 70%) of the desired product as an off-white waxy solid; TLC (Et_2O/CH_2Cl_2, 1:1) R_F = 0.42; $[\alpha]_D^{20}$ = -104.4 (1.00, $CHCl_3$); ^1H NMR (600 MHz, DMSO-d_6) δ: 9.72 (s, 1H), 8.10 (s, 1H), 7.87 (d, 1H, *J* = 8.0 Hz), 7.52-7.48 (m, 4H), 7.38-7.36 (m, 2H), 6.88-6.86 (m, 2H), 6.76 (s, 1H), 5.47-5.42 (m, 3H), 3.59-3.56 (m, 2H), 1.51 (d, 3H, *J* = 7.1 Hz), 0.84-0.81 (m, 2H), -0.10 (s, 9H); ^{13}C NMR (150 MHz, DMSO-d_6) δ: 157.6, 154.7, 151.4, 145.0, 137.2, 131.0 (2C), 129.9 (2C), 128.3 (2C), 122.4, 119.4, 115.6 (2C), 102.7, 98.1, 70.1, 65.6, 48.4, 25.5, 22.7, 17.3, -1.4 (3C); IR (neat, cm^{-1}): 3345, 3042, 2951, 2925, 1601, 1497, 1470, 1343, 1247, 1216, 1174, 1073, 1009, 858, 834, 769, 694; HRMS (ASAP-TOF, *m/z*): calcd. for $C_{26}H_{32}N_4O_2Si^{79}Br$, 539.1478 $[M+H]^+$, found 539.1473.

***(R)*-4-(4-((1-(4-Iodophenyl)ethyl)amino)-7-((2-(trimethylsilyl)ethoxy)methyl)-7*H*-pyrrolo[2,3-*d*]pyrimidin-6-yl)phenol (29)**

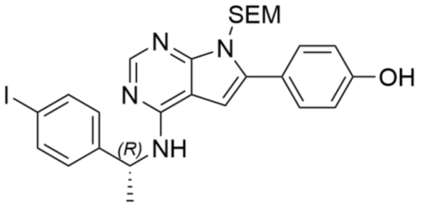

Compound **29** was made as described in the General Procedure A starting with **24** (93 mg, 0.248 mmol) and *(R)*-1-(4-iodophenyl)-ethylamine hydrochloride (227 mg, 0.801 mmol) and DIPEA (230 µL, 1.32 mmol). The resulting crude product was purified twice by flash chromatography (first C18 silica: MeCN/H_2O cont. 0.1% NEt_3, 3:1, then: silica-gel, EtOAc/*n*-pentane, 3:1). This gave 71 mg (0.121 mmol, 49%) of the desired product as a light brown solid, mp. 91.3-94.4 °C. TLC (EtOAc/*n*-pentane 1:3) R_f = 0.19; ^1H NMR (600 MHz, DMSO-d_6) δ: 9.71 (s, 1H), 8.10 (s, 1H), 7.86 (d, *J* = 7.9 Hz, 1H), 7.69 – 7.61 (m, 2H), 7.55 – 7.48 (m, 2H), 7.27 – 7.19 (m, 2H), 6.90 – 6.84 (m, 2H), 6.76 (s, 1H), 5.46 – 5.35 (m, 3H), 3.63 – 3.54 (m, 2H), 1.50 (d, *J* = 7.0 Hz, 3H), 0.86 – 0.76 (m, 2H), -0.10 (s, 9H); ^{13}C NMR (151 MHz, DMSO-d_6) δ: 157.6, 154.7, 151.4 (2C), 145.4, 137.2, 136.9 (2C), 129.9 (2C), 128.5 (2C), 122.4, 115.6 (2C), 102.7, 98.1, 92.1, 70.1, 65.6, 48.5, 22.6, 17.3, -1.4 (3C); IR (neat, cm^{-1}): 2950, 1600, 1497, 1469, 1242, 1265, 1215, 1074, 858, 833; HRMS (TOF ES+, *m/z*): calcd. for $C_{26}H_{32}N_4O_2SiI$ $[M+H]^+$: 587.1339, found: 587.1345.

***(R)*-4-(4-((1-(3-Bromophenyl)ethyl)amino)-7-((2-(trimethylsilyl)ethoxy)methyl)-7*H*-pyrrolo[2,3-*d*]pyrimidin-6-yl)phenol (30)**

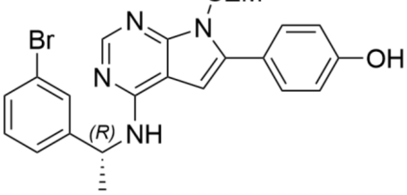

Compound **30** was prepared as described in General Procedure A starting from **24** (152 mg, 0.404 mmol) and (*R*)-1-(3-bromophenyl)ethan-1-amine (185 µL, 1.23 mmol). The crude product was immobilized on Celite and purified by gradient flash chromatography (silica, EtOAc/*n*-pentane, 0:1 to 1:3), resulting in 162 mg (0.301 mmol, 75%) of the desired product as a brown solid, mp. 147.4-148.5 °C; TLC (EtOAc/*n*-pentane 1:2) R_f = 0.19; ^1H NMR (600 MHz, DMSO-d_6) δ: 9.72

(s, 1H), 8.12 (s, 1H), 7.88 (d, *J* = 8.0 Hz, 1H), 7.61 (t, *J* = 1.9 Hz, 1H), 7.54 – 7.51 (m, 2H), 7.42 (d, *J* = 7.9 Hz, 1H), 7.41 – 7.38 (m, 1H), 7.27 (t, *J* = 7.8 Hz, 1H), 6.90 – 6.86 (m, 2H), 6.77 (s, 1H), 5.45 (s, 3H), 3.61 – 3.55 (m, 2H), 1.52 (d, *J* = 7.1 Hz, 3H), 0.82 (td, *J* = 7.6, 0.7 Hz, 2H), -0.10 (s, 9H); ^{13}C NMR (151 MHz, DMSO-d_6) δ: 157.6, 154.7, 151.4, 148.5, 137.3, 130.5, 129.9 (2C), 129.4, 128.7, 125.3, 122.4, 121.6, 115.6 (2C), 102.7 98.1, 70.2, 65.6, 48.5, 22.8, 17.3, -1.4 (3C); IR (neat, cm^{-1}): 3409, 2950, 1601, 1438, 1229, 1066, 835, 771, 693; HRMS (TOF ES+, *m/z*): calcd. for $C_{26}H_{32}N_4O_2Si^{79}Br$ [M+H]$^+$: 539.1478, found: 539.1481.

4-(4-((4-Bromobenzyl)amino)-7-((2-(trimethylsilyl)ethoxy)methyl)-7*H*-pyrrolo[2,3-*d*]pyrimidin-6-yl)phenol (31)

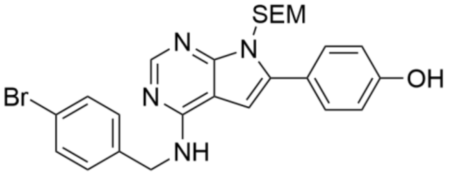

Compound **31** was prepared as described in General Procedure A, starting from **24** (150 mg, 0.399 mmol) and (4-bromophenyl)methanamine (155 µL, 1.23 mmol). The crude product was immobilized on Celite and purified by flash chromatography (silica, EtOAc/*n*-pentane 0:1 to 1:1) resulting in 164 mg (0.313 mmol, 78%) of the desired product as a brown wax, mp. 155.5-156.0 °C. TLC (EtOAc/*n*-pentane 1:2) R_f = 0.14; ^{1}H NMR (600 MHz, DMSO-d_6) δ: 9.72 (s, 1H), 8.17 (s, 1H), 8.10 (t, *J* = 6.1 Hz, 1H), 7.53 – 7.47 (m, 4H), 7.33 – 7.29 (m, 2H), 6.90 – 6.84 (m, 2H), 6.65 (s, 1H), 5.45 (s, 2H), 4.70 (d, *J* = 6.1 Hz, 2H), 3.62 – 3.56 (m, 2H), 0.86 – 0.80 (m, 2H), -0.09 (s, 9H); ^{13}C NMR (151 MHz, DMSO-d_6) δ: 157.6, 155.4, 151.5, 151.4, 139.7, 137.3, 131.1 (2C), 130.0 (2C), 129.4 (2C), 122.3, 119.6, 115.6 (2C), 102.7, 97.9, 70.2, 65.6, 42.5, 40.1, 17.3, -1.4 (3C); IR (neat, cm^{-1}): 3414, 2949, 1606, 1486, 1242, 1236, 1068, 834, 774; HRMS (TOF ES+, *m/z*): calcd. for $C_{25}H_{30}N_4O_2Si^{79}Br$ [M+H]$^+$: 525.1321, found: 525.1328.

(*S*)-4-(4-((1-(4-Bromophenyl)ethyl)amino)-7-((2-(trimethylsilyl)ethoxy)methyl)-7*H*-pyrrolo[2,3-*d*]pyrimidin-6-yl)phenol (32)

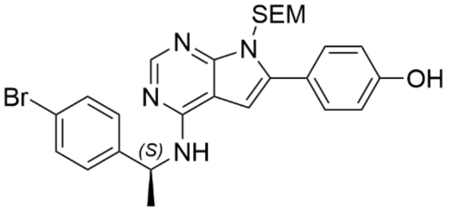

Compound **32** was prepared as described in General Procedure A, starting from **24** (153 mg, 0.407 mmol) and (*S*)-1-(4-bromophenyl)ethan-1-amine (174 µL, 1.20 mmol) The crude product was immobilized on Celite and purified by gradient flash chromatography (silica, EtOAc/*n*-pentane, 0:1 to 1:1), giving 159 mg (0.295 mmol, 72%) of the desired product as a brown solid, mp. 92.4-93.7 °C. TLC (EtOAc/*n*-pentane 1:2) R_f = 0.13; ^{1}H NMR (600 MHz, DMSO-d_6) δ: 8.23 (s, 1H), 7.56 – 7.51 (m, 4H), 7.28 (m, 2H), 6.85 (d, *J* = 8.6 Hz, 2H), 6.71 (s, 1H), 6.40 (s, 1H), 5.50 (s, 2H), 3.64 – 3.59 (m, 2H), 3.06 (s, 3H), 1.57 (d, *J* = 7.0 Hz, 3H), 0.88 – 0.83 (m, 2H), -0.07 (s, 9H); ^{13}C NMR (151 MHz, DMSO-d_6) δ: 157.6, 156.3, 152.9, 150.8, 141.0, 136.9, 131.3 (2C), 130.1 (2C), 129.2 (2C), 122.1, 120.1, 115.5 (2C), 102.4, 101.0, 70.2, 65.7, 51.9, 21.3, 17.4, -1.4 (3C); IR (neat, cm^{-1}): 2951, 1469, 1490, 1414, 1318, 1257, 1068, 1007, 832, 618; HRMS (TOF ES+, *m/z*): calcd. for $C_{26}H_{32}N_4O_2Si^{79}Br$ [M+H]$^+$: 539.1478, found: 539.1475.

(R)-4-(4-((1-(4-Bromophenyl)ethyl)(methyl)amino)-7-((2-(trimethylsilyl)ethoxy)methyl)-7*H*-pyrrolo[2,3-*d*]pyrimidin-6-yl)phenol (33)

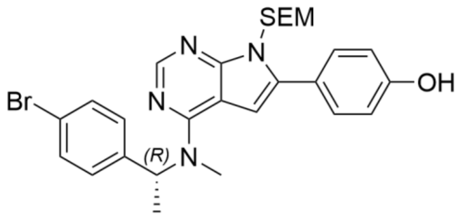

Compound **33** was prepared as described in General Procedure A, starting from **24** (190 mg, 0.505 mmol) and *(R)*-1-(4-bromophenyl)-*N*-methylethan-1-amine HCl-salt (401 mg, 1.60 mmol). The crude product was immobilized on Celite and purified by flash chromatography (silica, AcOH/EtOAc/*n*-pentane, 1:0:99 to 1:24:75), recovering 30% of the starting material and giving 89 mg, (0.161 mmol, 32%) of the desired product as a white solid, mp. 150.8-151.8 °C. TLC (AcOH/EtOAc/*n*-pentane 1:19:80) R_f = 0.24; ^{1}H NMR (600 MHz, DMSO-d_6) δ: 8.23 (s, 1H), 7.56 – 7.51 (m, 4H), 7.28 (m, 2H), 6.85 (d, *J* = 8.6 Hz, 2H), 6.71 (s, 1H), 6.40 (s, 1H), 5.50 (s, 2H), 3.64 – 3.59 (m, 2H), 3.06 (s, 3H), 1.57 (d, *J* = 7.0 Hz, 3H), 0.88 – 0.83 (m, 2H), -0.07 (s, 9H); ^{13}C NMR (151 MHz, DMSO-d_6) δ: 157.6, 156.3, 152.9, 150.8, 141.0, 136.9, 131.3 (2C), 130.1 (2C), 129.2 (2C), 122.1, 120.1, 115.5 (2C), 102.4, 101.0, 70.2, 65.7, 51.9, 21.3, 17.4, -1.4 (3C); IR (neat, cm^{-1}): 2951 (br, w), 1469, 1490, 1414, 1318, 1257, 1068, 1007, 832, 764, 618; HRMS (TOF ES+, *m/z*): calcd. for $C_{27}H_{34}N_4O_2Si^{79}Br$ $[M+H]^+$: 553.1634, found: 553.1634.

(*R*)-*N*-(1-(4-bromophenyl)ethyl)-6-(4-methoxyphenyl)-7-((2-(trimethylsilyl)ethoxy)meth-yl)-7*H*-pyrrolo[2,3-*d*]pyrimidin-4-amine (35)

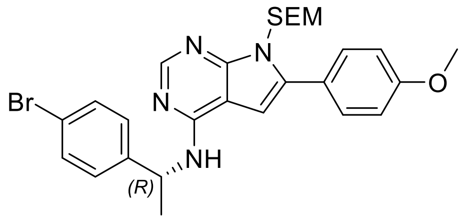

Compound **35** was prepared by Suzuki cross-coupling from **34** (205 mg, 0.358 mmol) and (4-methoxyphenyl)boronic acid as described in General Procedure B. The reaction time was 20 minutes. The crude material was purified by gradient flash chromatography (silica-gel, acetone/*n*-pentane, 0/100 to 1:4). TLC (acetone/*n*-pentane, 1:4) R_f = 0.33. This gave 146 mg (0.264 mmol, 74%) as a colorless oil; ^{1}H NMR (600 MHz, $CDCl_3$) δ: 8.35 (s, 1H), 7.63-7.62 (m, 2H), 7.45-7.44 (m, 2H), 7.31-7.30 (m, 2H), 6.99-6.98 (m, 2H), 6.34 (s, 1H), 5.52-5.48 (m, 3H), 5.25 (br s, 1H), 3.86 (s, 3H), 3.73-3.70 (m, 2H), 1.63 (d, 3H, *J* = 6.8 Hz), 0.96-0.93 (m, 2H), -0.04 (s, 9H); ^{13}C NMR (150 MHz, $CDCl_3$) δ: 160.0, 155.1, 152.4, 152.0, 143.4, 138.8, 131.9 (2C), 130.7 (2C), 128.0 (2C), 124.2, 121.1, 114.3 (2C), 103.1, 97.2, 70.7, 66.6, 55.5, 49.8, 23.1, 18.2, -1.3 (3C); IR (neat, cm^{-1}): 3284, 2952, 2897, 2836, 1600, 1498, 1467, 1250, 1212, 1179, 1160, 1143, 1076, 835, 601; HRMS (ASAP-TOF, *m*/*z*): calcd. for $C_{27}H_{34}N_4O_2{}^{79}BrSi$, 553.1634 $[M+H]^+$, found 553.1633

(*R*)-*N*-(1-(4-Bromophenyl)ethyl)-6-phenyl-7-((2-(trimethylsilyl)ethoxy)methyl)-7*H*-pyrrolo[2,3-*d*]pyrimidin-4-amine (36)

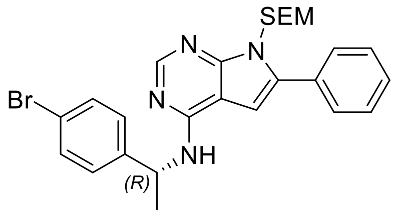

Compound **36** was synthesized as described in General Procedure B from **34** (100 mg, 0.174 mmol) and phenylboronic acid (21 mg, 0.172 mmol). The reaction time was 10 minutes. The crude material was purified by gradient flash chromatography (silica-gel, acetone/*n*-pentane, 0:100 to 1:6). This gave 63 mg (0.120 mmol, 69%) as a colorless oil; TLC (acetone/*n*-pentane, 1:6) R_f = 0.20; $[\alpha]_D^{20}$ = -92.8 (1.00, $CHCl_3$); ^{1}H NMR (600 MHz, $CDCl_3$) δ: 8.37 (s, 1H), 7.71-7.69 (m, 2H), 7.47-7.44 (m, 4H), 7.42-7.39 (m, 1H), 7.32-7.30 (m, 2H), 6.41 (s, 1H), 5.55 (s, 2H), 5.53-5.48 (m, 1H), 5.18 (br s, 1H), 3.73-3.70 (m, 2H), 1.64 (d, *J* = 6.9 Hz, 3H), 0.96-0.93 (m, 2H), -0.04 (s, 9H); ^{13}C NMR (150 MHz, $CDCl_3$)

δ: 155.3, 152.6, 152.3, 143.4, 138.9 (2C), 131.9, 131.8, 129.3 (2C), 128.8 (2C), 128.5, 128.0 (2C), 121.1, 103.1, 98.0, 70.8, 66.7, 49.8, 23.1, 18.2, -1.3 (3C); IR (neat, cm^{-1}): 3283, 3058, 3030, 2952, 2894, 1596, 1487, 1468, 1302, 1248, 1213, 1073, 859, 834, 755, 698; HRMS (ASAP-TOF, *m/z*): calcd. for $C_{26}H_{32}N_4OSi^{79}Br$, 523.1529 $[M+H]^+$, found: 523.1521

(*R*)-*N*-(1-(4-Bromophenyl)ethyl)-6-(4-fluorophenyl)-7-((2-(trimethylsilyl)ethoxy)meth-yl)-7*H*-pyrrolo[2,3-*d*]pyrimidin-4-amine (37)

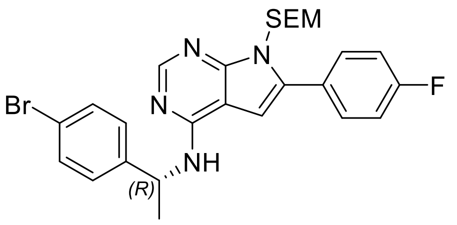

Compound **37** was made from **34** (100 mg, 0.174 mmol) and (4-fluorophenyl)boronic acid (25 mg, 0.179 mmol), as described in General Procedure B. Purification by flash chromatography (silica, acetone/*n*-pentane 1:5, R_f = 0.28) gave 63 mg (0.116 mmol, 67%) as a colorless oil; $[\alpha]_D^{20}$= -99.4 (1.00, $CHCl_3$); ^1H NMR (600 MHz, $CDCl_3$) δ: 8.36 (s, 1H), 7.70-7.67 (m, 2H), 7.47-7.45 (m, 2H), 7.31-7.30 (m, 2H), 7.16-7.13 (m, 2H), 6.37 (s, 1H), 5.53-5.48 (m, 3H), 5.16 (br d, 1H, *J* = 7.0 Hz), 3.74-3.71 (m, 2H), 1.64 (d, 3H, *J* = 6.9 Hz), 0.96-0.93 (m, 2H), -0.03 (s, 9H); ^{13}C NMR (150 MHz, $CDCl_3$) δ: 163.0 (d, *J* = 248.8 Hz), 155.3, 152.5, 152.4, 143.4, 137.8, 131.9 (2C), 131.2 (d, *J* = 7.9 Hz, 2C), 128.0 (2C), 127.9 (d, *J* = 3.3 Hz), 121.1, 115.9 (d, *J* = 21.8 Hz, 2C), 103.0, 98.0, 70.7, 66.7, 49.8, 23.0, 18.2, -1.3 (3C); ^{19}F NMR (376 MHz, $CDCl_3$, C_6F_6) δ: -116.2 (s); IR (neat, cm^{-1}): 3284, 3042, 2952, 2895, 1599, 1496, 1468, 1341, 1247, 1223, 1075, 1009, 858, 836, 766; HRMS (ASAP-TOF, *m/z*): calcd. for $C_{26}H_{31}N_4OSi^{79}Br$ F, 541.1435 $[M+H]^+$, found 541.1432.

(*R*)-*N*-(1-(4-Bromophenyl)ethyl)-6-(4-nitrophenyl)-7-((2-(trimethylsilyl)ethoxy)methyl)-7*H*-pyrrolo[2,3-*d*]pyrimidin-4-amine (38)

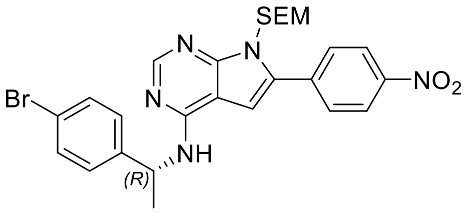

Compound **38** was made from **34** (300 mg, 0.523 mmol) and (4-nitrophenyl)boronic acid (87 mg, 0.521 mmol) as described in General Procedure B. The reaction time was 10 minutes. Purification by flash chromatography (silica, acetone/*n*-pentane, 1:4, R_F = 0.23) gave 186 mg (0.327 mmol, 63%) of a yellow-red wax; $[\alpha]_D^{20}$= -178.0 (1.00, $CHCl_3$); ^1H NMR (600 MHz, $CDCl_3$) δ: 8.37 (s, 1H), 8.31-8.29 (m, 2H), 7.94-7.92 (m, 2H), 7.47-7.45 (m, 2H), 7.32-7.30 (m, 2H), 6.59 (s, 1H), 5.59-5.48 (m, 4H), 3.80-3.77 (m, 2H), 1.65 (d, *J* = 6.7 Hz, 3H), 0.99-0.96 (m, 2H), -0.02 (s, 9H); ^{13}C NMR (150 MHz, $CDCl_3$) δ: 155.6, 153.3, 153.1, 147.4, 143.1, 138.2, 136.2, 132.0 (2C), 129.4 (2C), 127.9 (2C), 124.2 (2C), 121.3, 103.0, 100.8, 70.9, 67.0, 50.1, 23.0, 18.2, -1.3 (3C); IR (neat, cm^{-1}): 3405, 3276, 3102, 3042, 2952, 2895, 1591, 1534, 1342, 1075, 1010, 856, 835, 751; HRMS (ASAP-TOF, *m/z*): calcd. for $C_{26}H_{31}N_5O_3Si^{79}Br$, 568.1380 $[M+H]^+$, found 568.1385.

Methyl (*R*)-4-(4-((1-(4-bromophenyl)ethyl)amino)-7-((2-(trimethylsilyl)ethoxy)methyl)-7*H*-pyrrolo[2,3-*d*]pyrimidin-6-yl)benzoate (39)

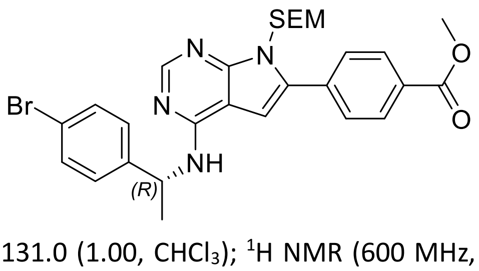

Compound **39** was prepared from **34** (301 mg, 0.525 mmol) and (4-(methoxycarbonyl)phenyl)boronic acid (94 mg, 0.522 mmol), as described in General Procedure B. The reaction time was 25 minutes. Purification by flash chromatography (silica, acetone/*n*-pentane, 1:9, R_f = 0.04) gave 170 mg (0.292 mmol, 56%) of a white wax; $[\alpha]_D^{20}$ = -131.0 (1.00, $CHCl_3$); ^1H NMR (600 MHz, $CDCl_3$) δ: 8.37 (s, 1H), 8.13-8.11 (m, 2H), 7.82-7.80 (m, 2H), 7.48-7.45 (m, 2H), 7.32-7.30 (m, 2H), 6.52 (s, 1H), 5.56 (s, 2H), 5.51 (quint., 1H, *J* = 6.9 Hz), 5.21 (br s,

1H), 3.95 (s, 3H), 3.76-3.73 (m, 2H), 1.64 (d, *J* = 6.9 Hz, 3H), 0.97-0.95 (m, 2H), -0.03 (s, 9H); ^{13}C NMR (150 MHz, $CDCl_3$) δ: 166.9, 155.4, 152.9, 152.8, 143.2, 137.7, 136.2, 131.9 (2C), 130.1 (2C), 129.8, 128.9 (2C), 128.0 (2C), 121.2, 103.2, 99.3, 70.9, 66.8, 52.4, 49.9, 23.0, 18.2, -1.2 (3C); IR (neat, cm^{-1}): 3378, 3277, 2951 2894, 1723, 1598, 1563, 1468, 1279, 1249, 1188, 1105, 1076, 860, 835, 772; HRMS (ASAP-TOF, *m/z*): calcd. for $C_{28}H_{34}N_4O_3{}^{79}BrSi$, 581.1584 $[M+H]^+$, found 581.1588.

(*R*)-4-(4-((1-(4-bromophenyl)ethyl)amino)-7-((2-(trimethylsilyl)ethoxy)methyl)-7*H*-pyrrolo[2,3-*d*]pyrimidin-6-yl)benzenesulfonamide (40)

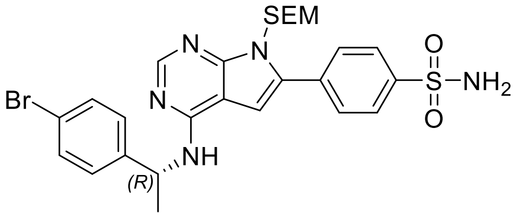

Compound **40** was prepared from **34** (200 mg, 0.349 mmol) and (4-sulfamoylphenyl)boronic acid (70 mg, 0.348 mmol) as described in General Procedure B. The reaction time was 10 minutes. Purification by gradient flash chromatography (silica-gel, acetone/*n*-pentane, 0:100 to 1:2); TLC (silica, acetone/*n*-pentane, 1:2) R_f = 0.43. This gave 136 mg (0.226 mmol, 65%) of a white wax; $[\alpha]_D^{20}$ = -145.4 (1.00, $CHCl_3$); ^{1}H NMR (600 MHz, $CDCl_3$) δ: 8.36 (s, 1H), 8.00 – 7.98 (m, 2H), 7.89 – 7.87 (m, 2H), 7.47 – 7.44 (m, 2H), 7.32 – 7.30 (m, 2H), 6.55 (s, 1H), 5.54 (s, 2H), 5.52 – 5.49 (m, 1H), 5.43 (br s, 1H), 5.08 (s, 2H), 3.77 – 3.75 (m, 2H), 1.65 (d, *J* = 6.8 Hz, 3H), 0.97 – 0.94 (m, 2H), -0.03 (s, 9H); ^{13}C NMR (150 MHz, $CDCl_3$) δ: 155.6, 153.0 (2C)*, 143.2, 141.3, 136.6, 136.4, 131.9 (2C), 129.5 (2C), 128.0 (2C), 127.0 (2C), 121.2, 103.1, 100.1, 70.8, 66.9, 49.9, 22.4, 18.2, -1.2 (3C), * 2C overlap seen by HMBC; IR (neat, cm^{-1}): 3372, 3259, 3020, 2952, 2895, 1593, 1562, 1470, 1338, 1306, 1157, 1072, 834, 751, 603; HRMS (ASAP-TOF, *m/z*): calcd. for $C_{26}H_{33}N_5O_3S^{79}BrSi$, 602.1257 $[M+H]^+$, found 602.1257.

(*R*)-2-(4-((1-(4-Bromophenyl)ethyl)amino)-7-((2-(trimethylsilyl)ethoxy)methyl)-7H-pyrrolo[2,3-d]pyrimidin-6-yl)phenol (41)

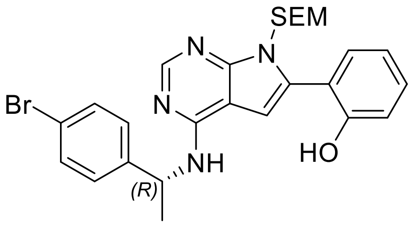

Compound **41** was prepared from **34** (200 mg, 0.349 mmol) and (2-hydroxyphenyl)boronic acid (48 mg, 0.348 mmol) as described in General Procedure B. Purification was by two rounds of flash chromatography (first: silica-gel EtOAc/*n*-pentane, 1:1, R_f= 0.45, then: C18 silica, acetone H_2O, 2:1, TLC (EtOAc/*n*-pentane, 1:1) R_f = 0.45). This gave 49 mg (0.091 mmol, 26%) as an off-white wax; $[\alpha]_D^{20}$ = -88.8 (1.00, $CHCl_3$); ^{1}H NMR (600 MHz, $CDCl_3$) δ: 8.35 (s, 1H), 7.47-7.44 (m, 2H), 7.38-7.35 (m, 1H), 7.31-7.28 (m, 3H),7.20-7.06 (m, 2H), 7.01 (td, *J* = 7.5, 1.2 Hz, 1H), 6.36 (s, 1H), 5.49-5.43 (m, 3H), 5.21-5.19 (m, 1H), 3.68-3.65 (m, 2H), 1.63 (d, *J* = 6.8 Hz, 3H), 0.94-0.91 (m, 2H), -0.05 (s, 9H); ^{13}C NMR (150 MHz, $CDCl_3$) δ: 155.3, 154.9, 152.6, 152.0, 143.2, 133.7, 132.4, 131.9 (2C), 131.2, 128.0 (2C), 121.2, 121.0, 119.2, 118.1, 103.2, 100.1, 70.8, 67.3, 49.9, 23.1, 18.0, -1.4 (3C); IR (neat, cm^{-1}): 3353, 3274, 3045, 2952, 2929, 1601, 1565, 1471, 1451, 1343, 1075, 859, 835, 756; HRMS (ASAP-TOF, *m/z*): calcd. for $C_{26}H_{32}N_4O_2{}^{79}BrSi$, 539.1478 $[M+H]^+$, found 539.1479.

(*R*)-3-(4-((1-(4-bromophenyl)ethyl)amino)-7-((2-(trimethylsilyl)ethoxy)methyl)-7*H*-pyrrolo[2,3-*d*]pyrimidin-6-yl)phenol (42)

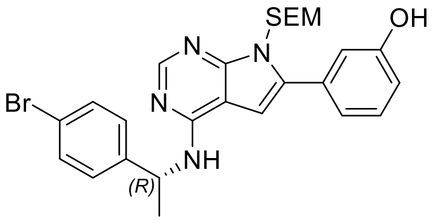

Compound **42** was synthesized from **34** (100 mg, 0.174 mmol) and (3-hydroxyphenyl)boronic acid (24 mg, 0.174 mmol) as described in General Procedure B. Purification by flash chromatography (silica, acetone/*n*-pentane, 1:2, R_f = 0.57) gave 50 mg (0.093 mmol, 53%) of an off-white wax; $[\alpha]_D^{20}$ = -92.0 (1.00, $CHCl_3$); ^{1}H NMR (600 MHz, $CDCl_3$) δ: 8.35 (s, 1H), 7.43-7.40 (m, 2H), 7.30-7.26 (m, 3H), 7.22-7.20 (m, 1H), 7.16-7.15 (m, 1H), 6.89-6.87 (ddd, *J* = 8.1, 2.5, 1.0 Hz, 1H), 6.39 (s, 1H), 5.54 (s, 2H), 5.49-5.44 (m, 1H), 5.40 (br s, 1H), 3.69-3.66 (m, 2H), 1.62 (d, *J* = 6.7 Hz, 3H), 0.92-0.87 (m, 2H), -0.07 (s, 9H) (OH-signal not observed); ^{13}C NMR (150 MHz, $CDCl_3$) δ: 156.6, 155.2, 152.3, 152.0, 143.2, 138.8, 133.0, 131.9 (2C), 130.1, 127.9 (2C), 121.4, 121.1, 116.3, 115.9, 103.1, 98.4, 70.9, 66.7, 50.1, 23.3, 18.1, -1.3 (3C); IR (neat, cm^{-1}): 3333, 3015, 2952, 2929, 2894, 1598, 1564, 1471, 1345, 1215, 1073, 858, 833, 752; HRMS (ASAP-TOF, *m*/*z*): calcd. for $C_{26}H_{32}N_4O_2{}^{79}BrSi$, 539.1478 $[M+H]^+$, found 539.1479.

NMR spectroscopy

Compound 1

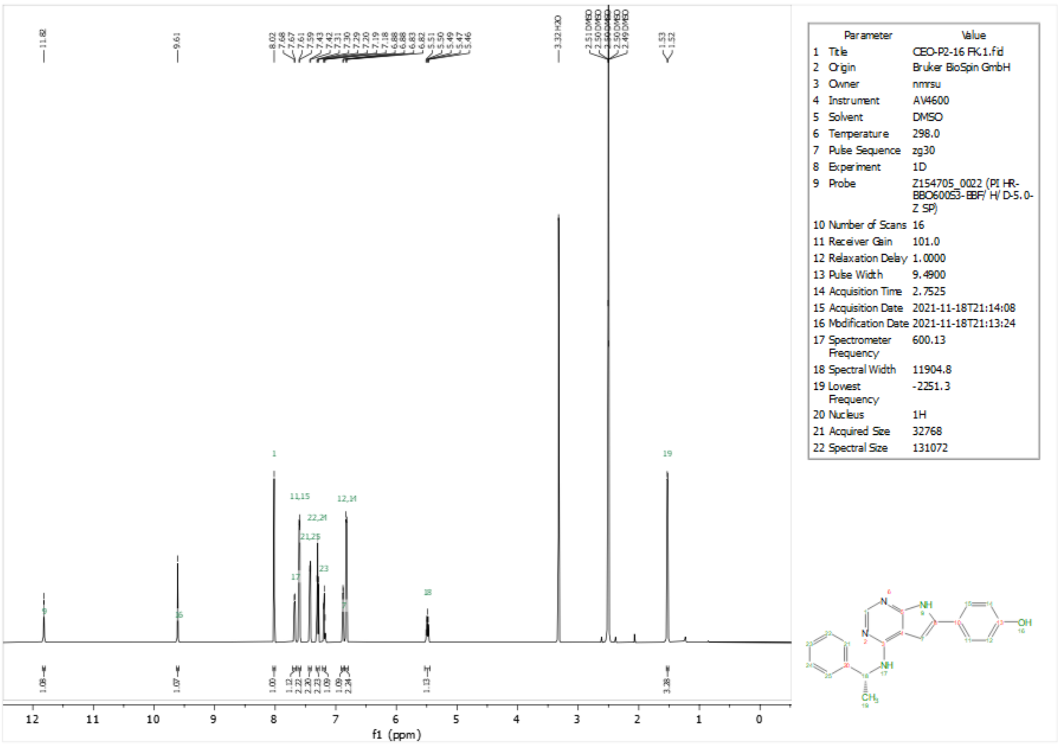

*Figure S1: ^{1}H NMR spectrum of compound **1** at 600 MHz in DMSO-d$_6$*

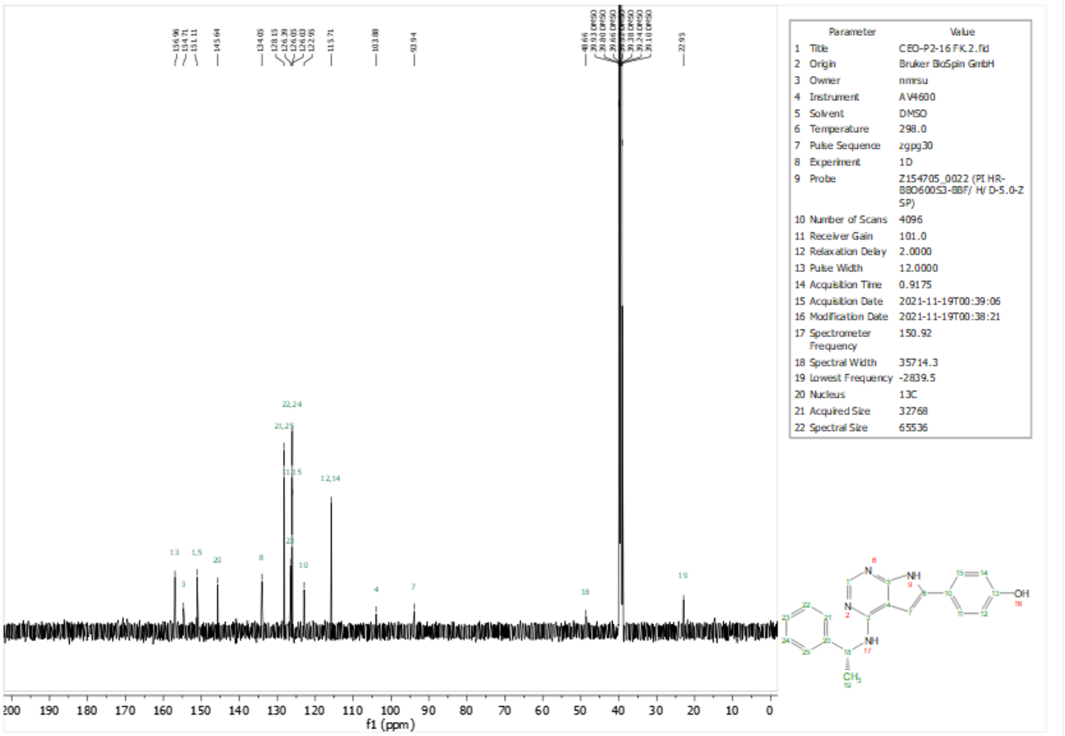

*Figure S2: ^{13}C NMR spectrum of compound **1** at 150 MHz in DMSO-d$_6$*

Compound 2

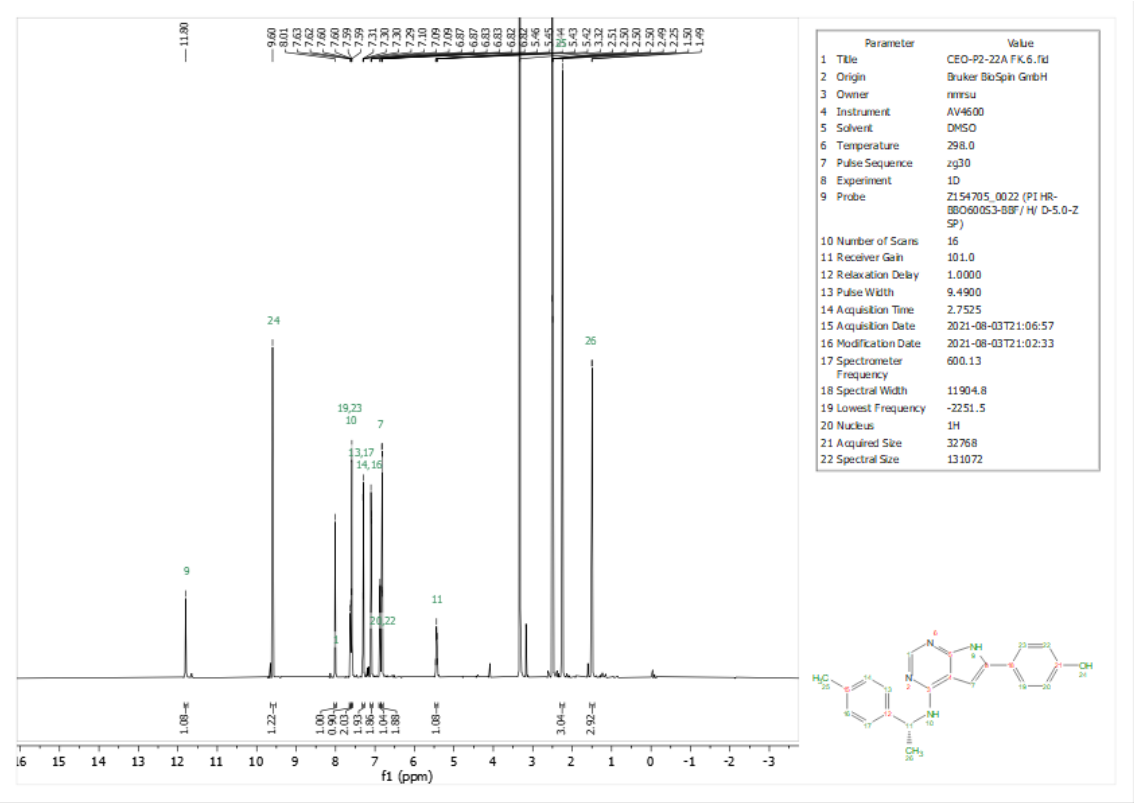

*Figure S3: ^{1}H NMR spectrum of compound **2** at 600 MHz in DMSO-d_6*

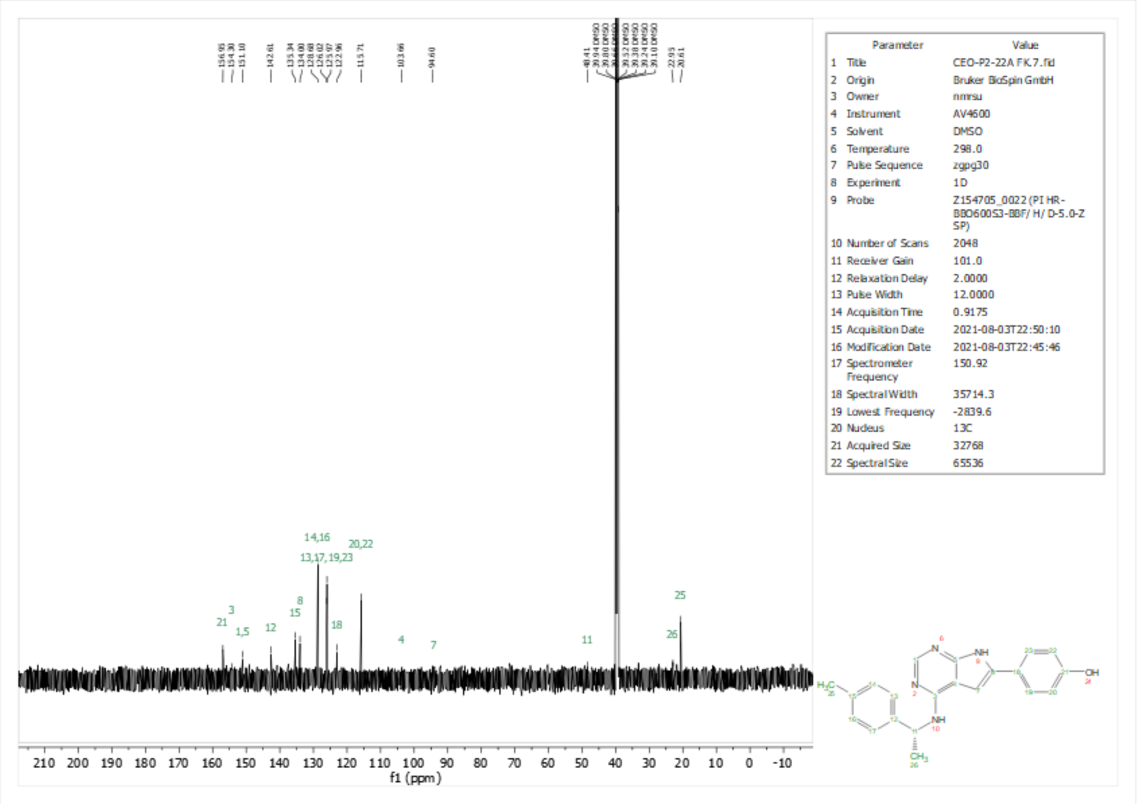

*Figure S4: ^{13}C NMR spectrum of compound **2** at 150 MHz in DMSO-d_6*

Compound 4

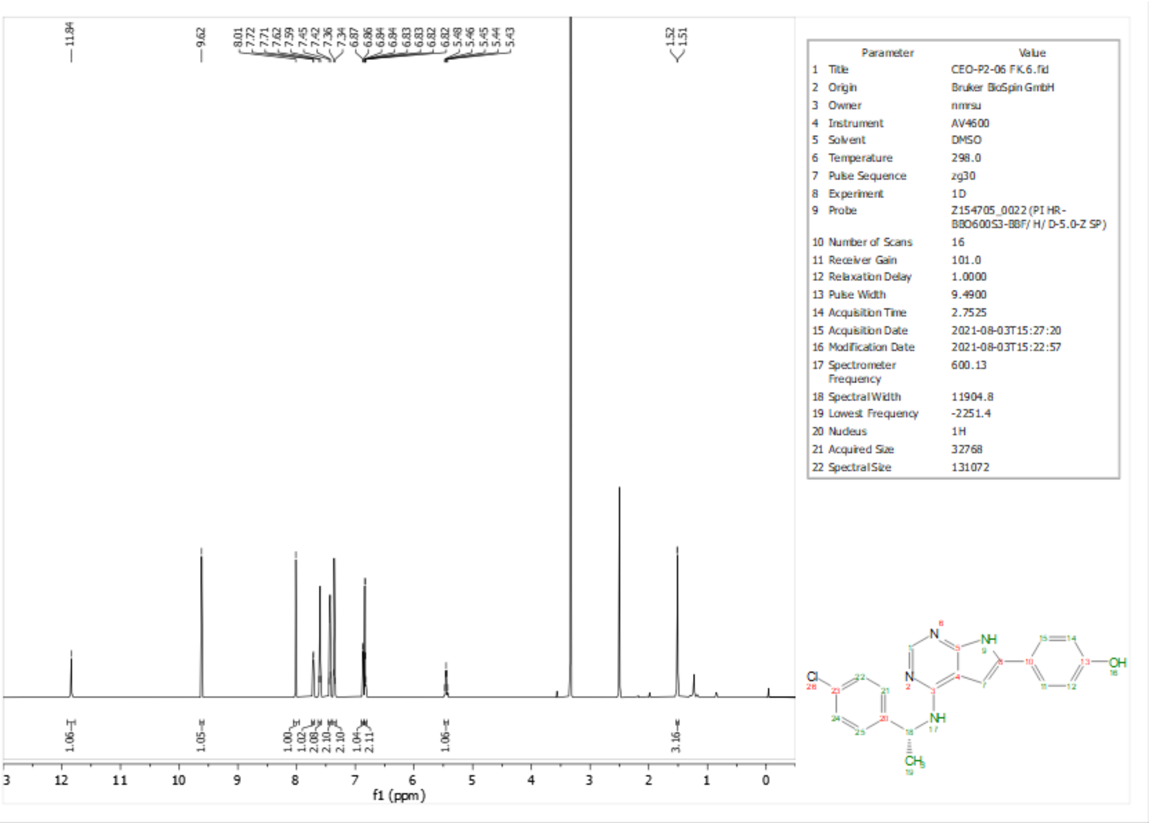

*Figure S5: ^{1}H NMR spectrum of compound **4** at 600 MHz in DMSO-d_6*

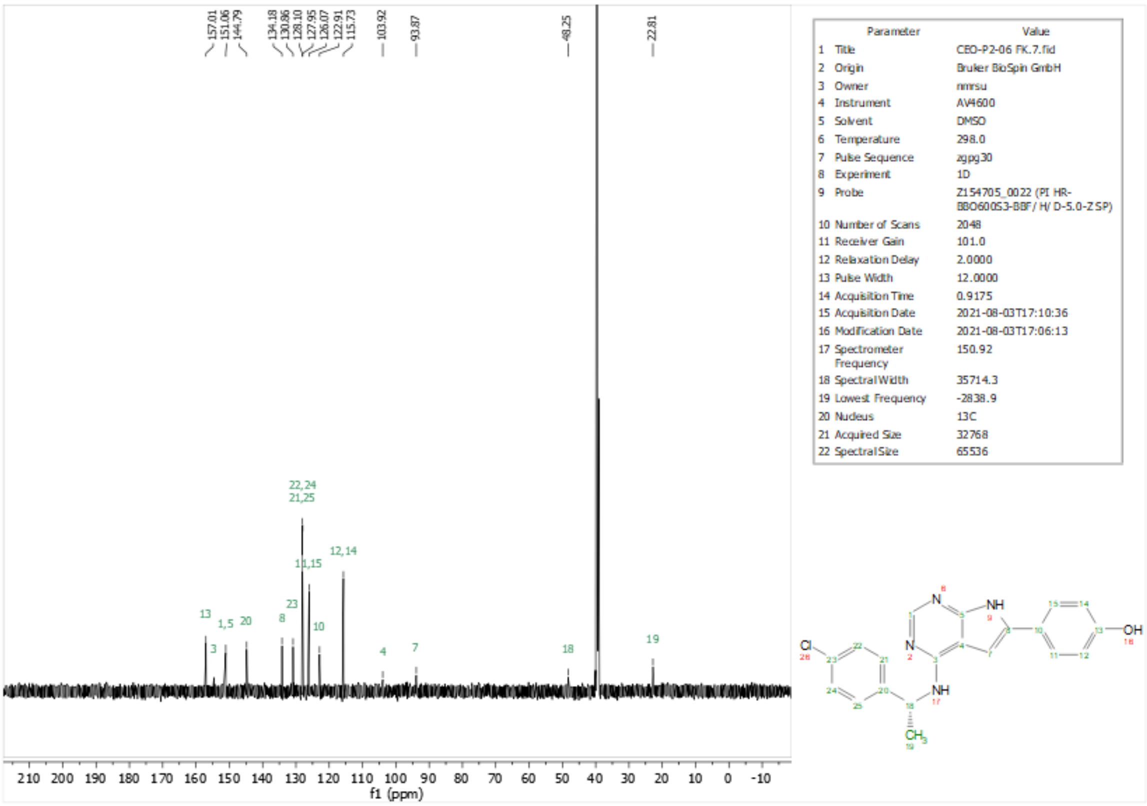

*Figure S6: ^{13}C NMR spectrum of compound **4** at 150 MHz in DMSO-d_6*

Compound 5

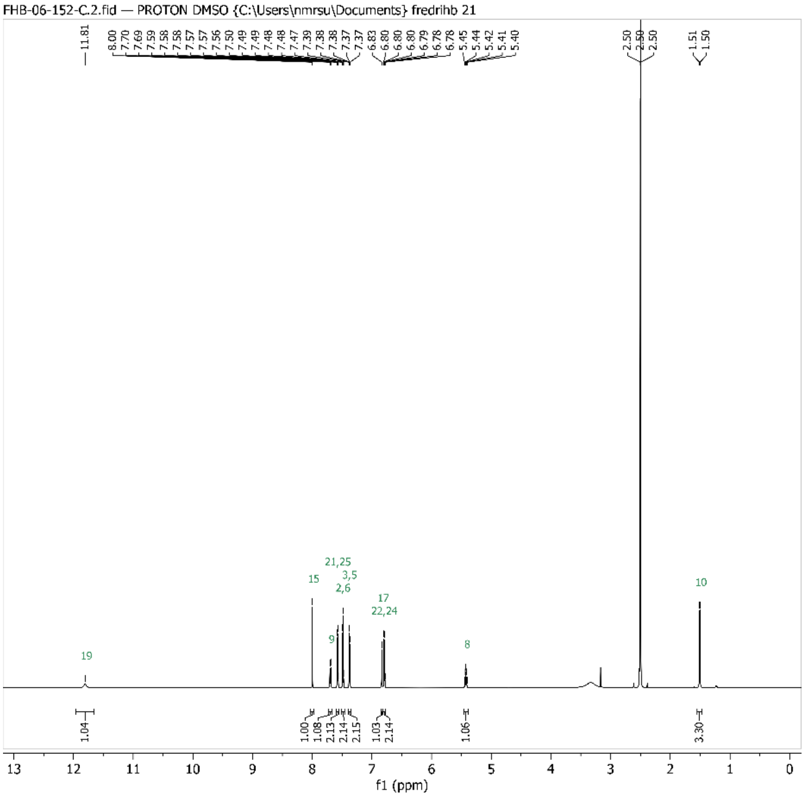

Parameter	Value
1 Origin	Bruker BioSpin GmbH
2 Owner	nmrsu
3 Instrument	spect
4 Solvent	DMSO
5 Temperature	298.0
6 Pulse Sequence	zg30
7 Experiment	1D
8 Probe	Z117768_0061 (CP TCI 600S3 H-C/ N-D-05 Z)
9 Number of Scans	16
10 Receiver Gain	11.5
11 Relaxation Delay	1.0000
12 Pulse Width	8.0000
13 Presaturation Frequency	
14 Acquisition Time	2.7263
15 Acquisition Date	2021-04-13T09:40:50
16 Modification Date	2021-04-13T09:40:50
17 Spectrometer Frequency	600.18
18 Spectral Width	12019.2
19 Lowest Frequency	-2303.5
20 Nucleus	1H
21 Acquired Size	32768
22 Spectral Size	131072
23 Digital Resolution	0.09

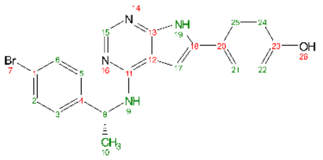

*Figure S7: ¹H NMR spectrum of compound **5** at 600 MHz in DMSO-d₆*

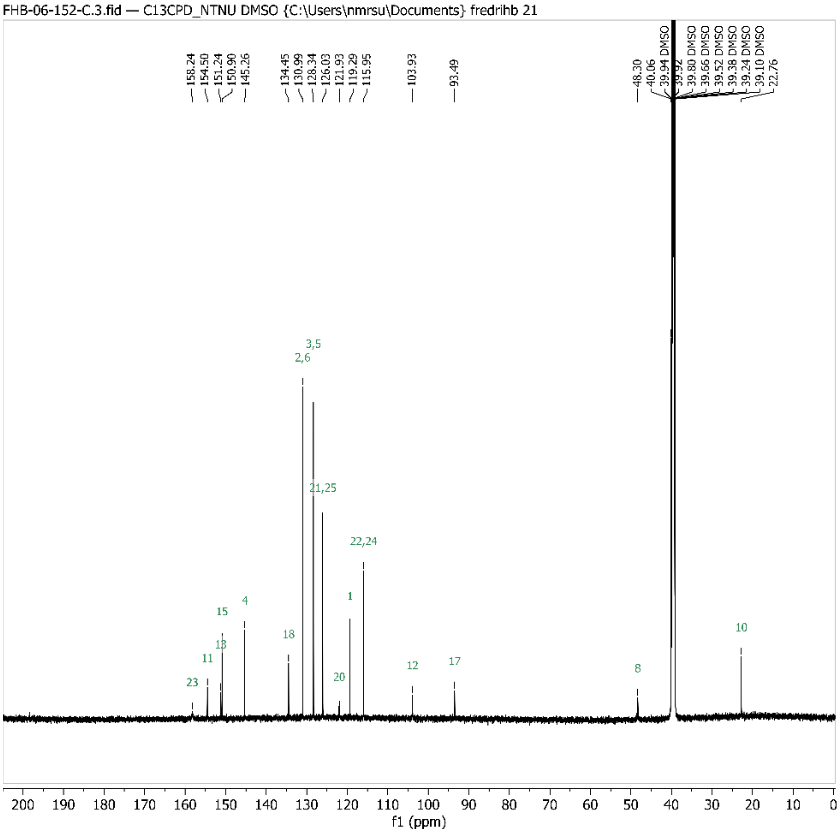

Parameter	Value
1 Origin	Bruker BioSpin GmbH
2 Owner	nmrsu
3 Instrument	spect
4 Solvent	DMSO
5 Temperature	300.0
6 Pulse Sequence	zgpg30
7 Experiment	1D
8 Probe	Z117768_0061 (CP TCI 600S3 H-C/ N-D-05 Z)
9 Number of Scans	2048
10 Receiver Gain	197.1
11 Relaxation Delay	2.0000
12 Pulse Width	11.4000
13 Presaturation Frequency	
14 Acquisition Time	0.9088
15 Acquisition Date	2021-04-13T11:24:37
16 Modification Date	2021-04-13T11:24:37
17 Spectrometer Frequency	150.93
18 Spectral Width	36057.7
19 Lowest Frequency	-3012.3
20 Nucleus	13C
21 Acquired Size	32768
22 Spectral Size	65536
23 Digital Resolution	0.55

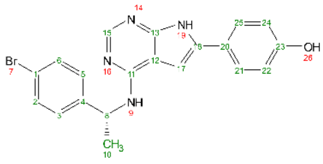

*Figure S8: ¹³C NMR spectrum of compound **5** at 150 MHz in DMSO-d₆*

Compound 6

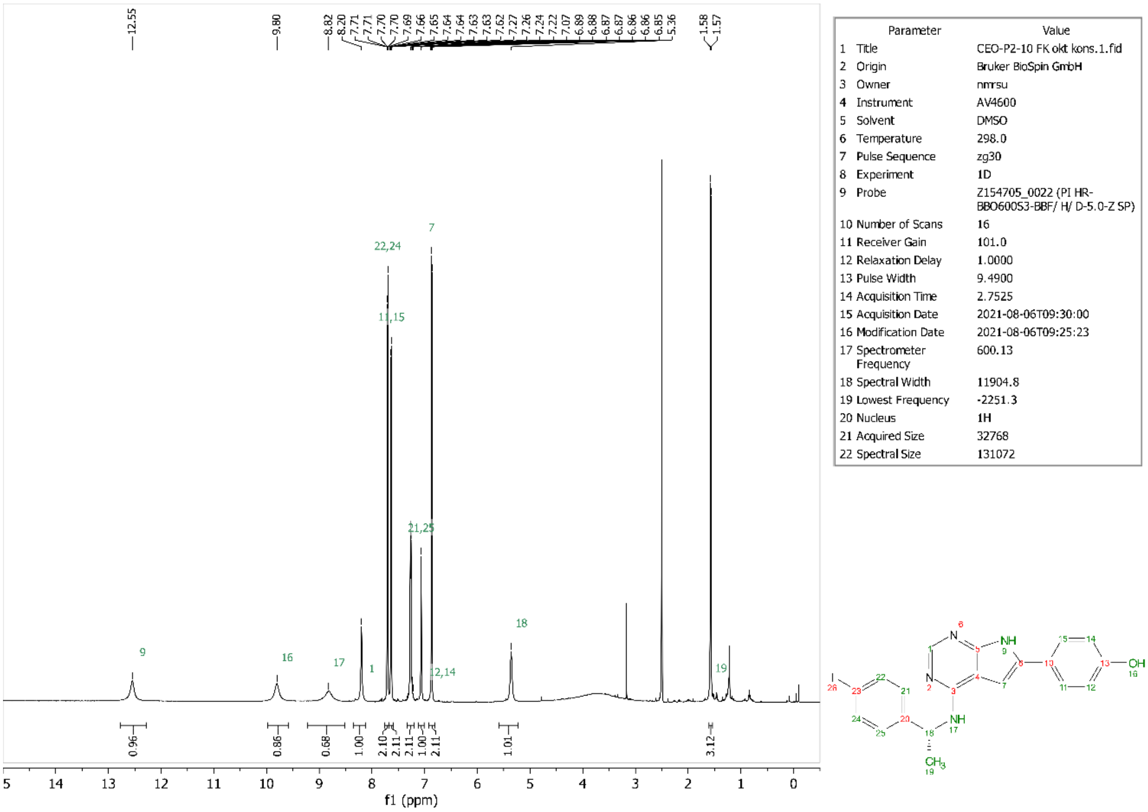

*Figure S9: ^{1}H NMR spectrum of compound **6** at 600 MHz in DMSO-d_6*

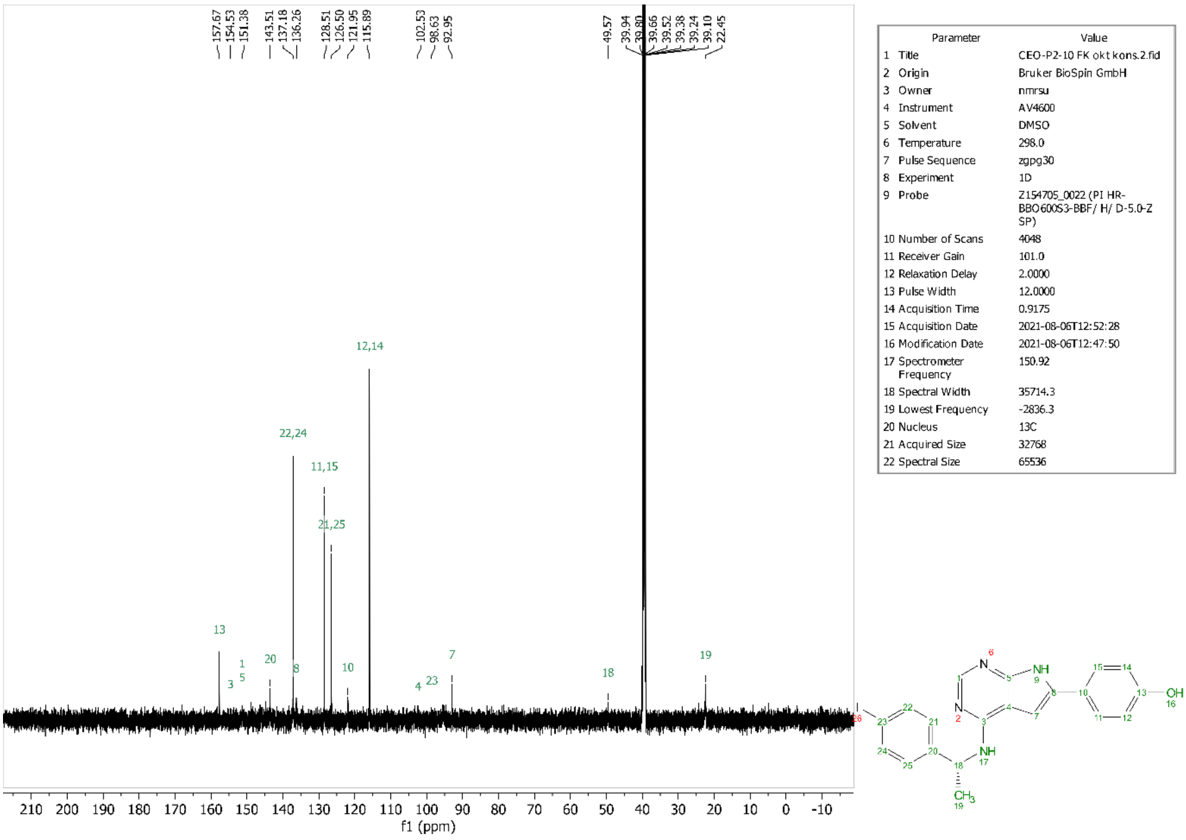

*Figure S10: ^{13}C NMR spectrum of compound **6** at 150 MHz in DMSO-d_6*

Compound 7

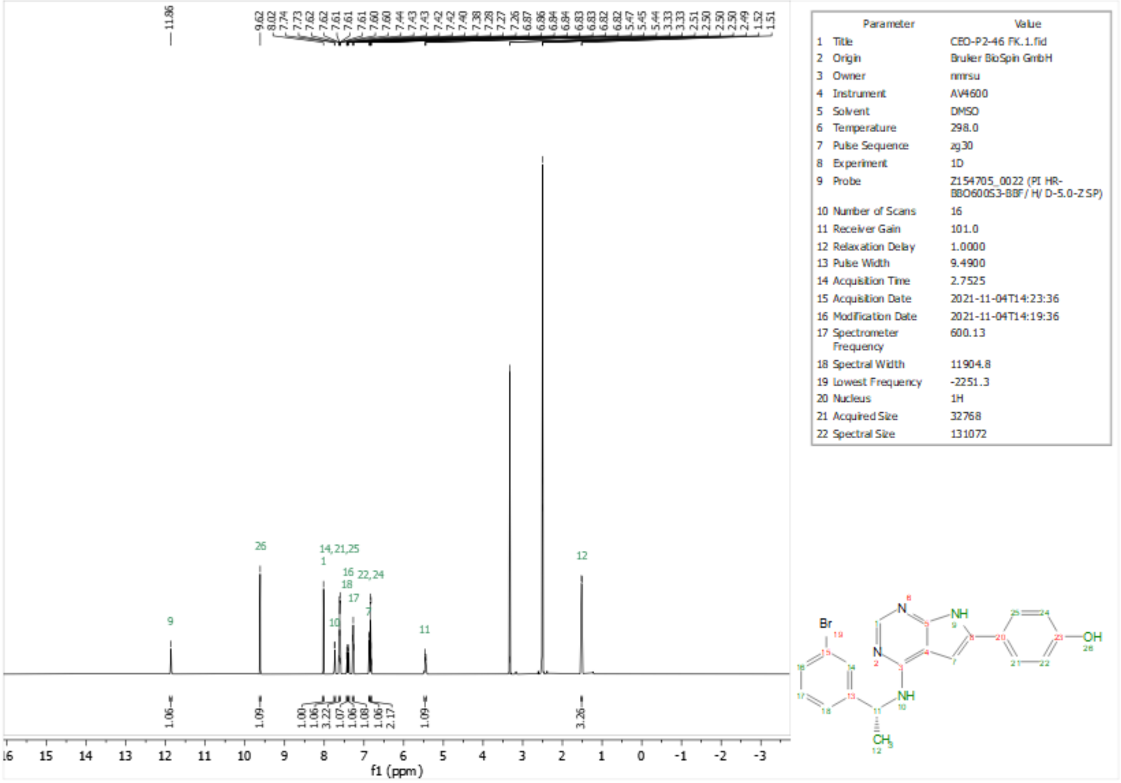

*Figure S11: ¹H NMR spectrum of compound **7** at 600 MHz in DMSO-d₆*

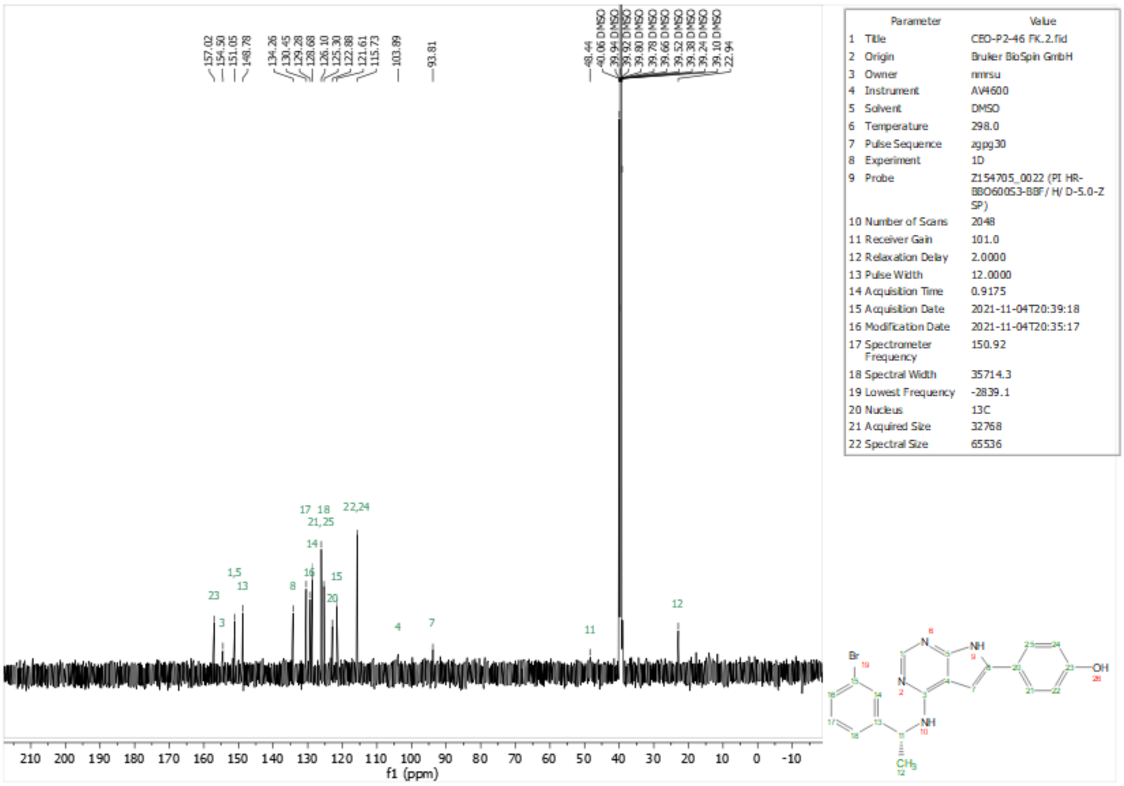

*Figure S12: ¹³C NMR spectrum of compound **7** at 150 MHz in DMSO-d₆*

Compound 8

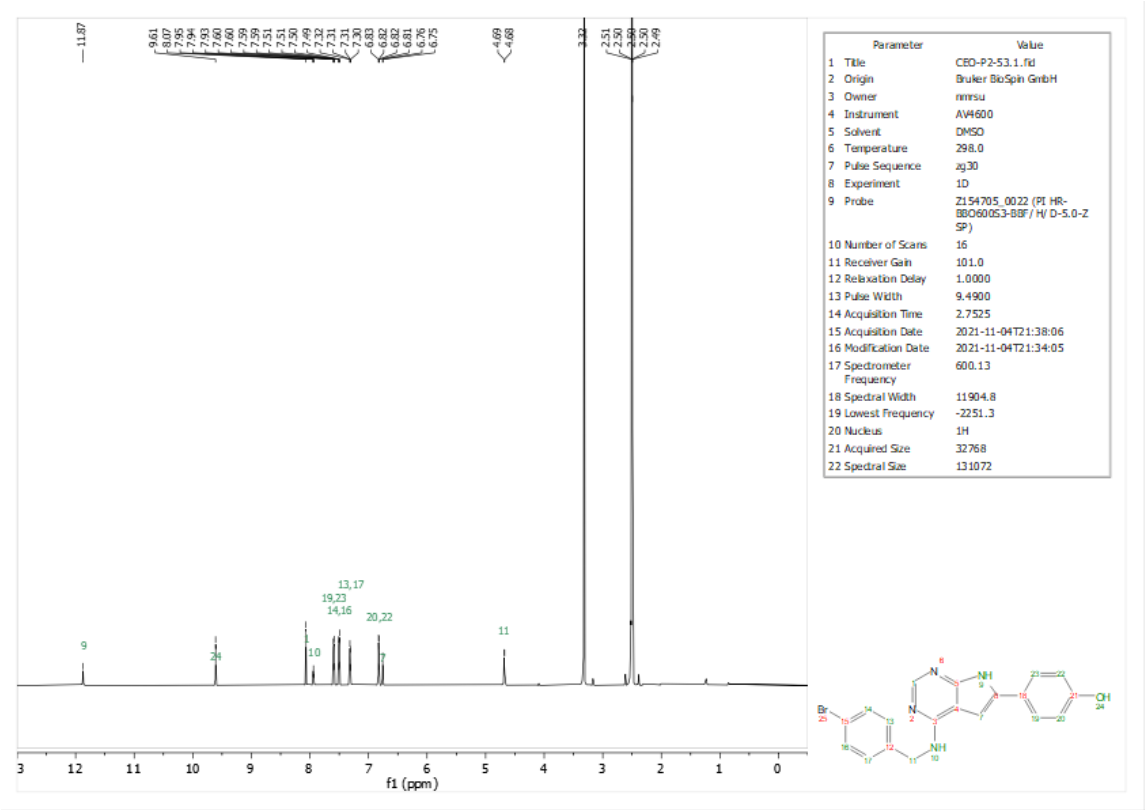

*Figure S13: ^{1}H NMR spectrum of compound **8** at 600 MHz in DMSO-d_6*

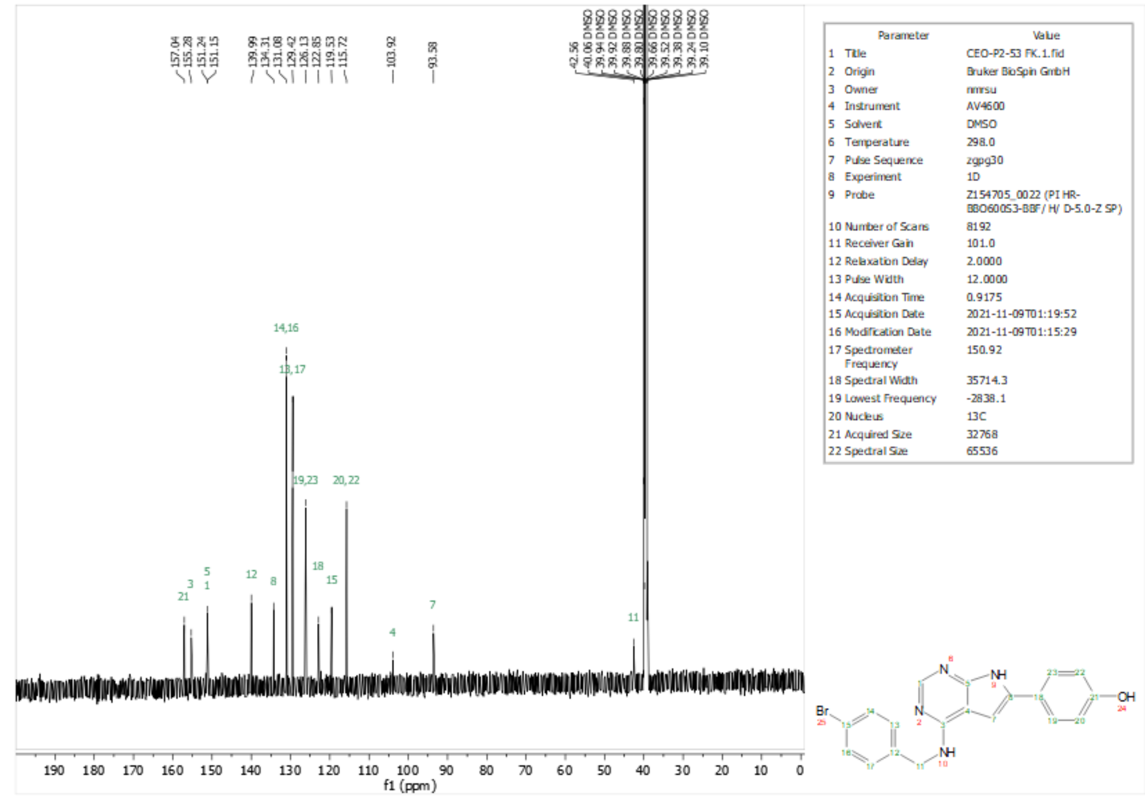

*Figure S14: ^{13}C NMR spectrum of compound **8** at 150 MHz in DMSO-d_6*

Compound 9

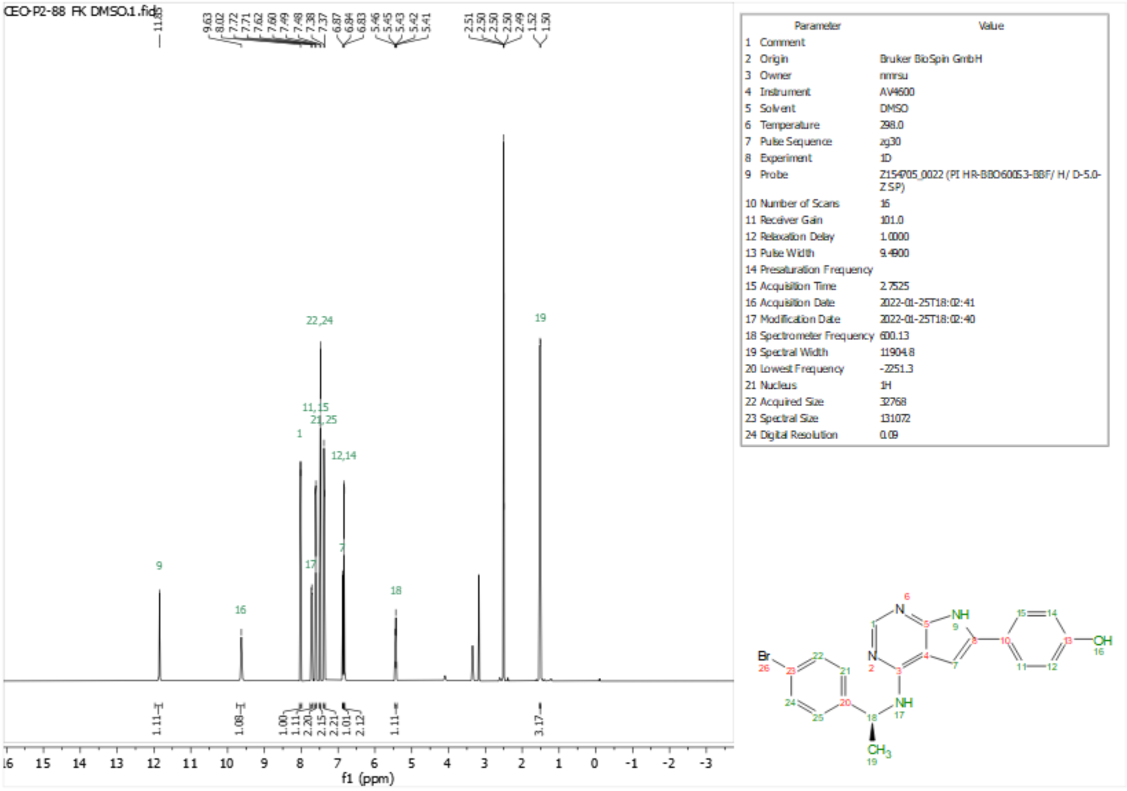

*Figure S15: *

Figure S15: ^{1}H NMR spectrum of compound **9** at 600 MHz in DMSO-d_6

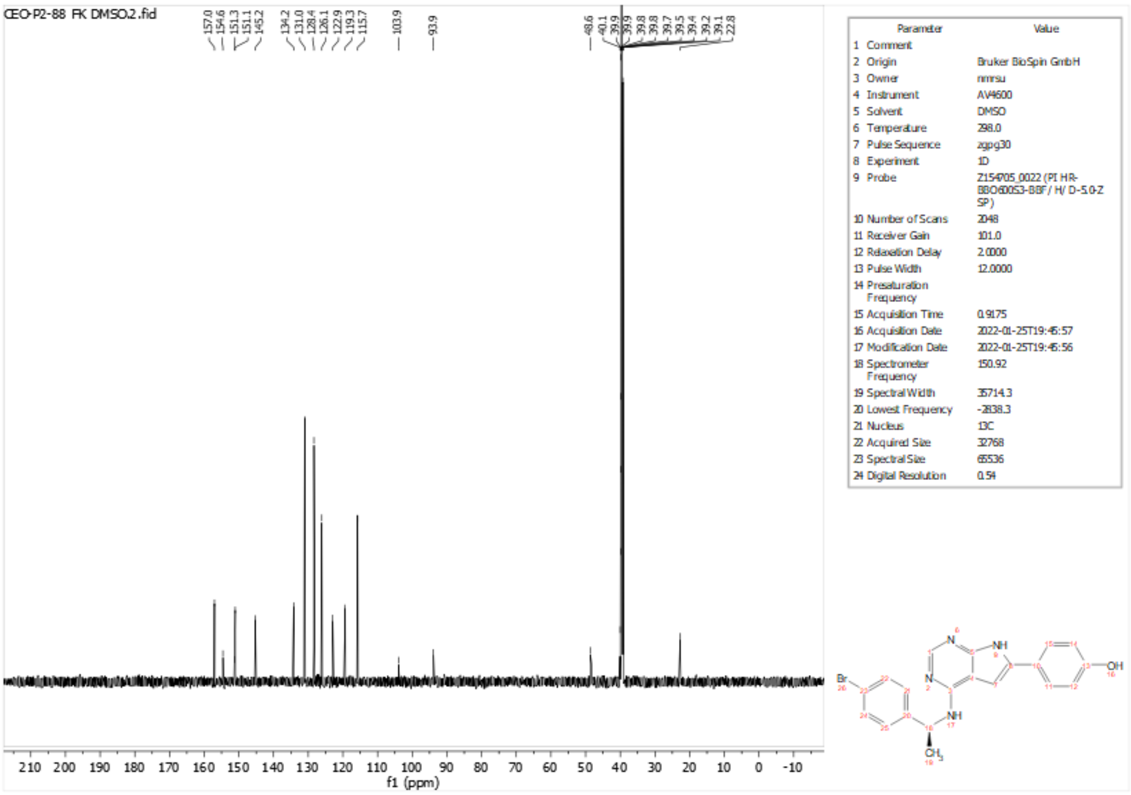

*Figure S16: ^{13}C NMR spectrum of compound **8** at 150 MHz in DMSO-d_6*

Compound 10

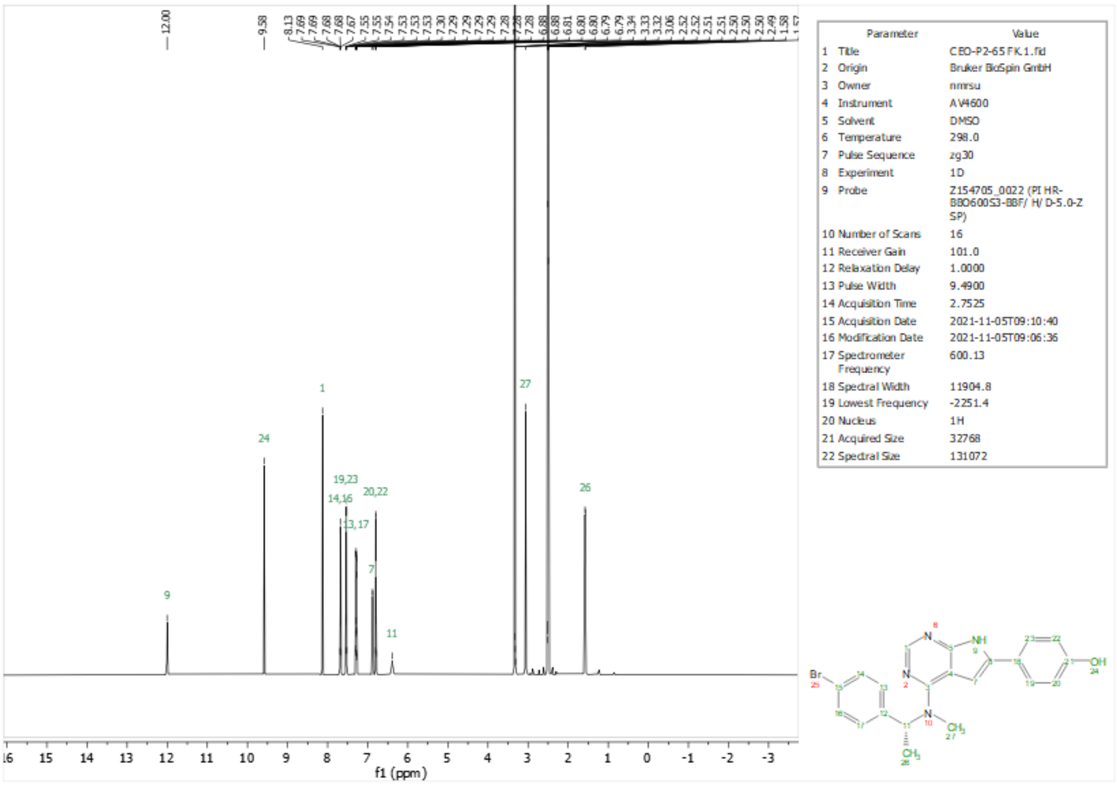

*Figure S17: ¹H NMR spectrum of compound **10** at 600 MHz in DMSO-d₆*

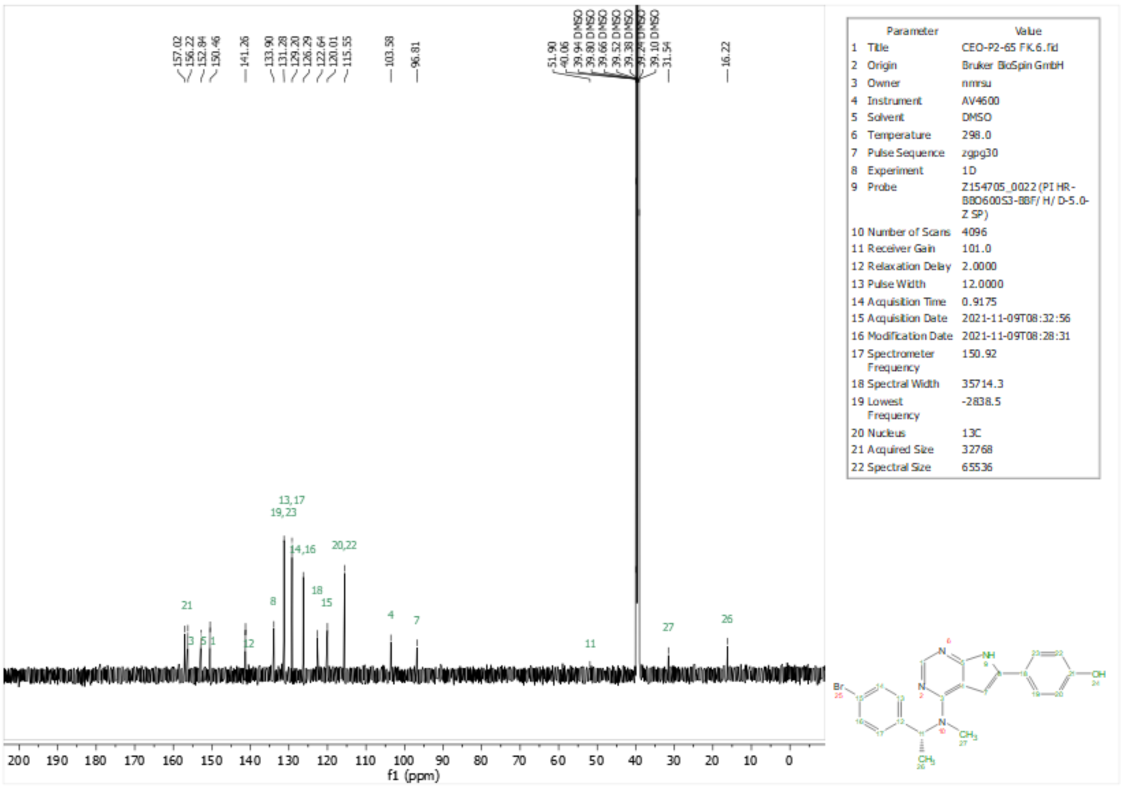

*Figure S18: ¹³C NMR spectrum of compound **10** at 150 MHz in DMSO-d₆*

Compound 11

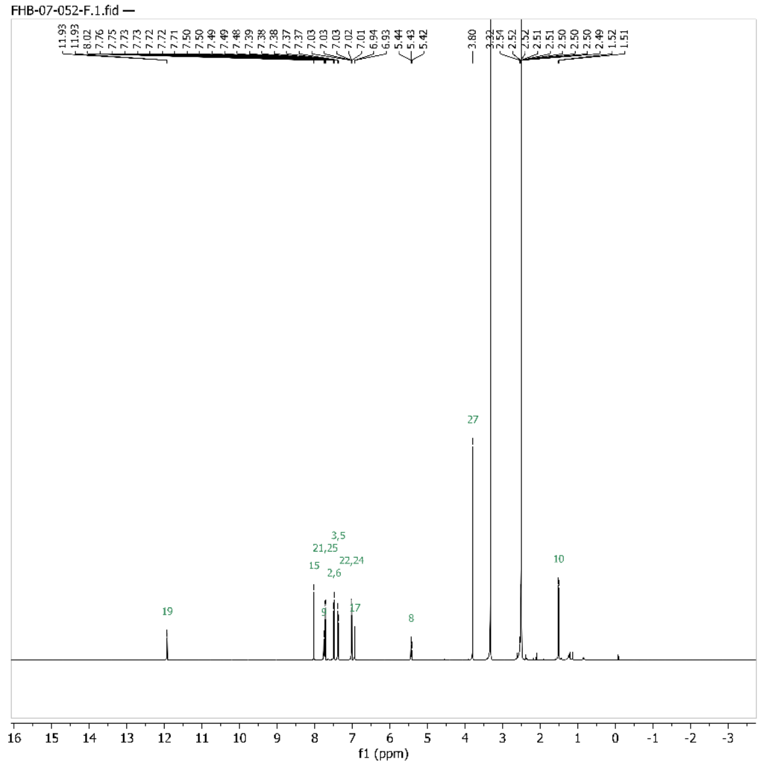

	Parameter	Value
1	Origin	Bruker BioSpin GmbH
2	Owner	nmrsu
3	Instrument	AV4600
4	Solvent	DMSO
5	Temperature	298.0
6	Pulse Sequence	zg30
7	Experiment	1D
8	Probe	Z154705_0022 (PI HR-BBO600S3-BBF/ H/ D-5.0-Z SP)
9	Number of Scans	16
10	Receiver Gain	101.0
11	Relaxation Delay	1.0000
12	Pulse Width	9.4900
13	Presaturation Frequency	
14	Acquisition Time	2.7525
15	Acquisition Date	2021-10-11T14:00:13
16	Modification Date	2021-10-11T13:58:21
17	Spectrometer Frequency	600.13
18	Spectral Width	11904.8
19	Lowest Frequency	-2211.9
20	Nucleus	1H
21	Acquired Size	32768
22	Spectral Size	131072
23	Digital Resolution	0.09

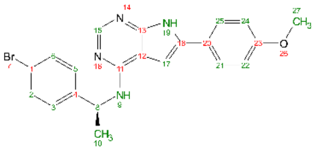

*Figure S19: ¹H NMR spectrum of compound **11** at 600 MHz in DMSO-d₆*

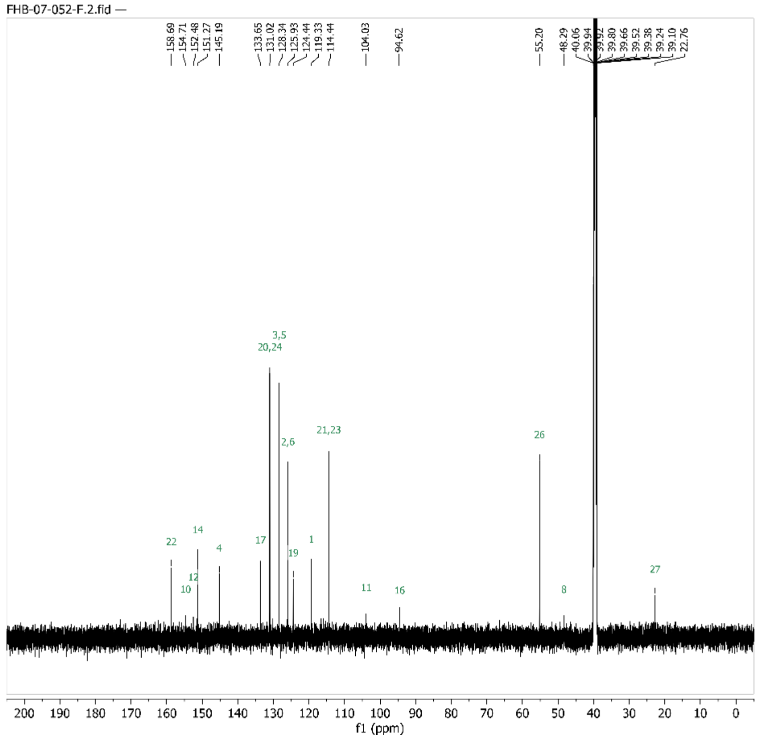

	Parameter	Value
1	Origin	Bruker BioSpin GmbH
2	Owner	nmrsu
3	Instrument	AV4600
4	Solvent	DMSO
5	Temperature	298.0
6	Pulse Sequence	zgpg30
7	Experiment	1D
8	Probe	Z154705_0022 (PI HR-BBO600S3-BBF/ H/ D-5.0-Z SP)
9	Number of Scans	2048
10	Receiver Gain	101.0
11	Relaxation Delay	2.0000
12	Pulse Width	12.0000
13	Presaturation Frequency	
14	Acquisition Time	0.9175
15	Acquisition Date	2021-10-11T15:43:30
16	Modification Date	2021-10-11T15:41:38
17	Spectrometer Frequency	150.92
18	Spectral Width	35714.3
19	Lowest Frequency	-2839.5
20	Nucleus	13C
21	Acquired Size	32768
22	Spectral Size	65536
23	Digital Resolution	0.54

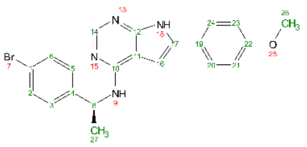

*Figure S20: ¹³C NMR spectrum of compound **11** at 150 MHz in DMSO-d₆*

Compound 12

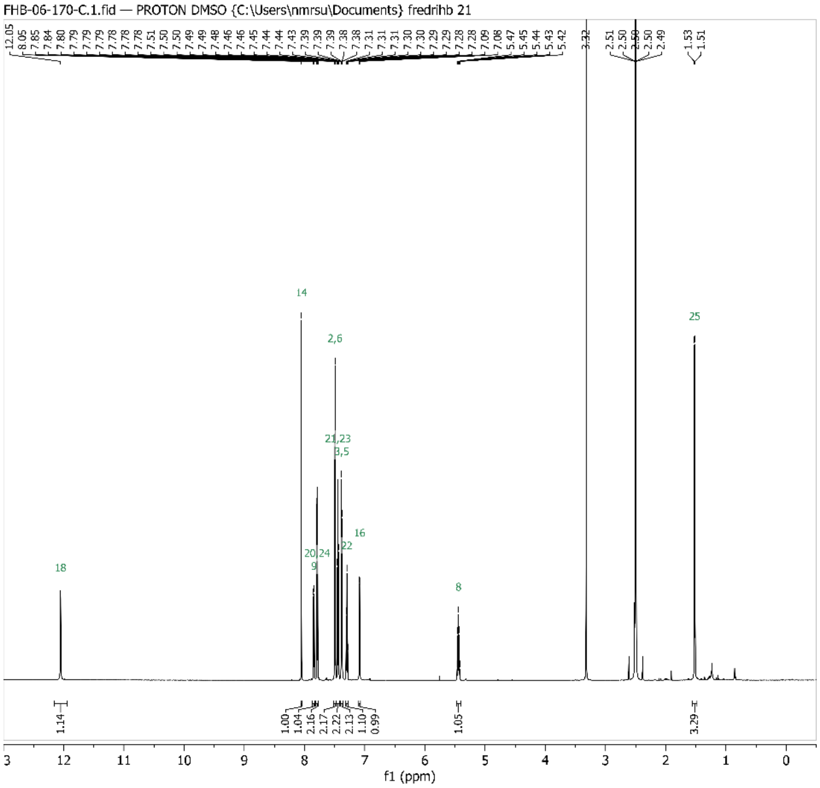

	Parameter	Value
1	Origin	Bruker BioSpin GmbH
2	Owner	nmrsu
3	Instrument	spect
4	Solvent	DMSO
5	Temperature	298.0
6	Pulse Sequence	zg30
7	Experiment	1D
8	Probe	Z117768_0061 (CP TCI 600S3 H-C/ N-D-05 Z)
9	Number of Scans	16
10	Receiver Gain	11.5
11	Relaxation Delay	1.0000
12	Pulse Width	8.0000
13	Presaturation Frequency	
14	Acquisition Time	2.7263
15	Acquisition Date	2021-04-27T21:05:23
16	Modification Date	2021-04-27T21:05:23
17	Spectrometer Frequency	600.18
18	Spectral Width	12019.2
19	Lowest Frequency	-2303.5
20	Nucleus	1H
21	Acquired Size	32768
22	Spectral Size	131072
23	Digital Resolution	0.09

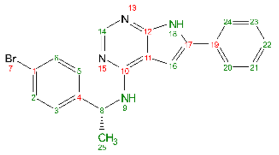

*Figure S21: ¹H NMR spectrum of compound **12** at 600 MHz in DMSO-d₆*

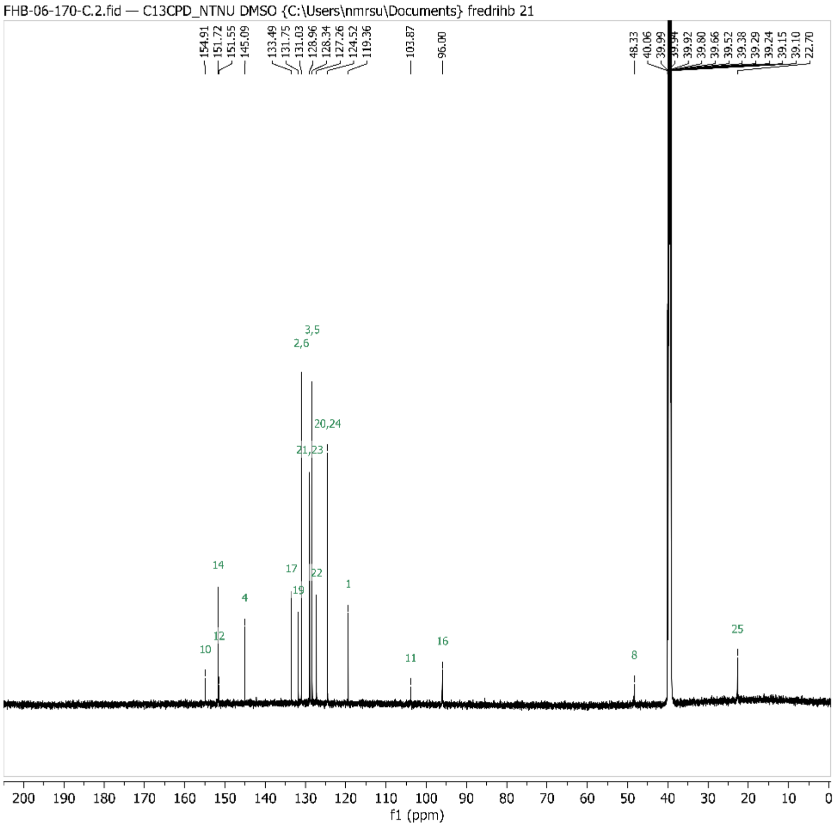

	Parameter	Value
1	Origin	Bruker BioSpin GmbH
2	Owner	nmrsu
3	Instrument	spect
4	Solvent	DMSO
5	Temperature	300.0
6	Pulse Sequence	zgpg30
7	Experiment	1D
8	Probe	Z117768_0061 (CP TCI 600S3 H-C/ N-D-05 Z)
9	Number of Scans	2048
10	Receiver Gain	197.1
11	Relaxation Delay	2.0000
12	Pulse Width	11.4000
13	Presaturation Frequency	
14	Acquisition Time	0.9088
15	Acquisition Date	2021-04-27T22:49:37
16	Modification Date	2021-04-27T22:49:38
17	Spectrometer Frequency	150.93
18	Spectral Width	36057.7
19	Lowest Frequency	-2938.0
20	Nucleus	13C
21	Acquired Size	32768
22	Spectral Size	65536
23	Digital Resolution	0.55

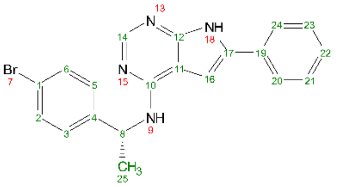

*Figure S22: ¹³C NMR spectrum of compound **12** at 150 MHz in DMSO-d₆*

Compound 13

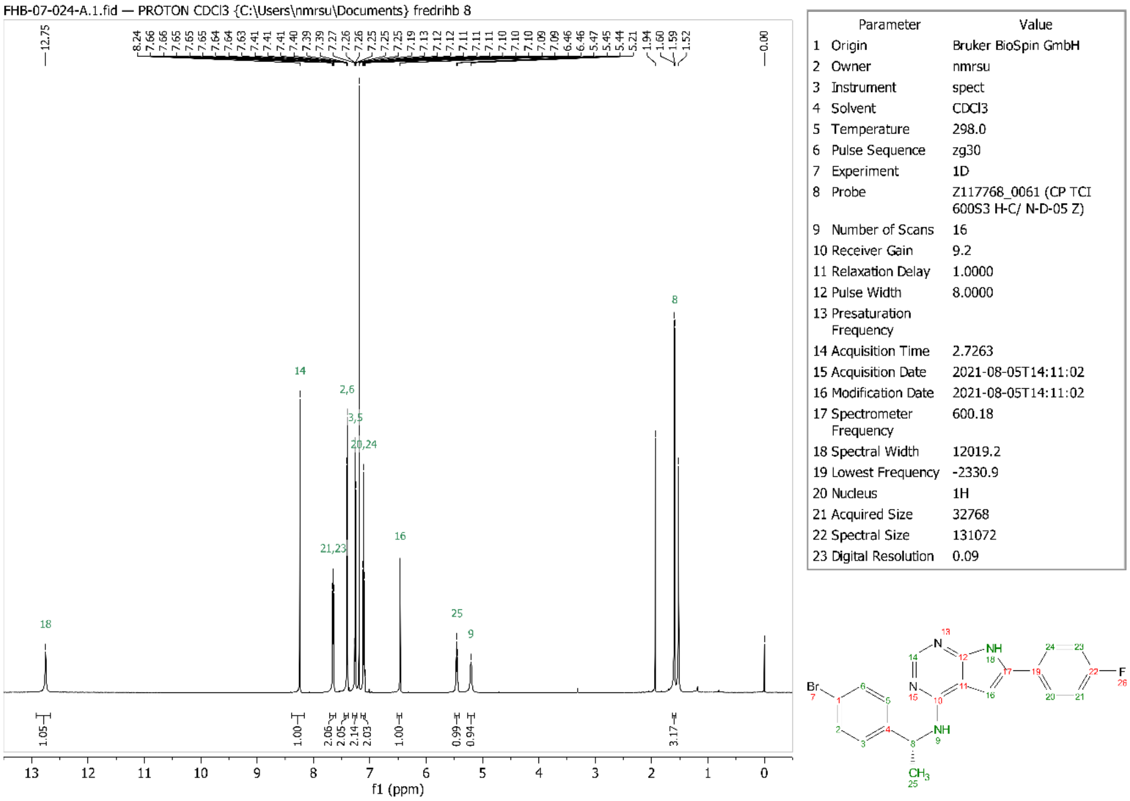

*Figure S23: ¹H NMR spectrum of compound **13** at 600 MHz in CDCl₃*

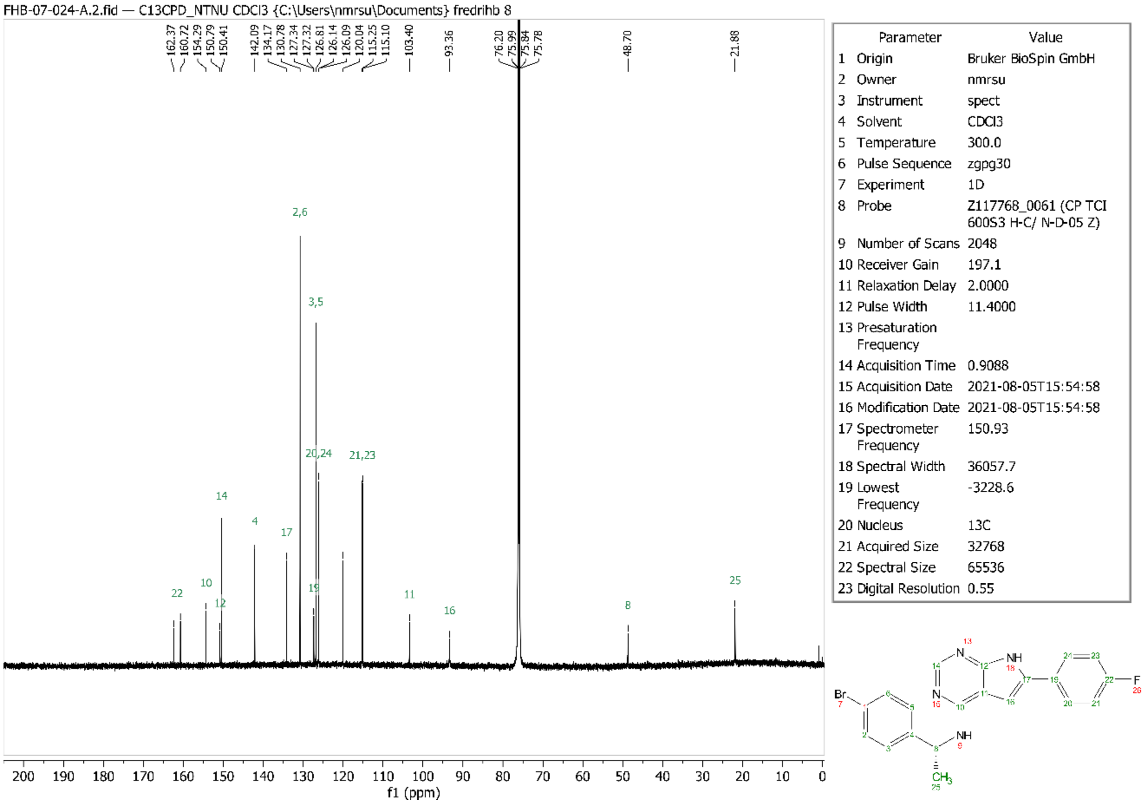

*Figure S24: ¹³C NMR spectrum of compound **13** at 150 MHz in CDCl₃*

Compound 14

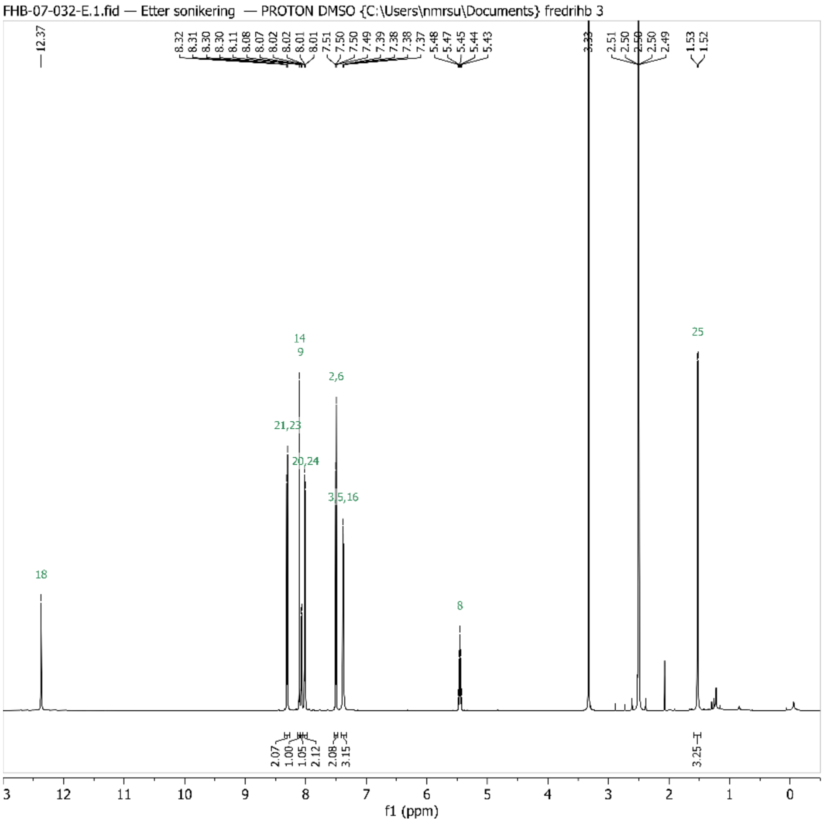

	Parameter	Value
1	Origin	Bruker BioSpin GmbH
2	Owner	nmrsu
3	Instrument	spect
4	Solvent	DMSO
5	Temperature	298.0
6	Pulse Sequence	zg30
7	Experiment	1D
8	Probe	Z117768_0061 (CP TCI 600S3 H-C/ N-D-05 Z)
9	Number of Scans	16
10	Receiver Gain	9.2
11	Relaxation Delay	1.0000
12	Pulse Width	8.0000
13	Presaturation Frequency	
14	Acquisition Time	2.7263
15	Acquisition Date	2021-08-13T09:10:44
16	Modification Date	2021-08-13T09:10:44
17	Spectrometer Frequency	600.18
18	Spectral Width	12019.2
19	Lowest Frequency	-2308.6
20	Nucleus	1H
21	Acquired Size	32768
22	Spectral Size	131072
23	Digital Resolution	0.09

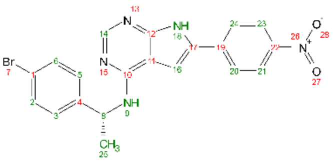

*Figure S25: ^{1}H NMR spectrum of compound **14** at 600 MHz in DMSO-d$_6$*

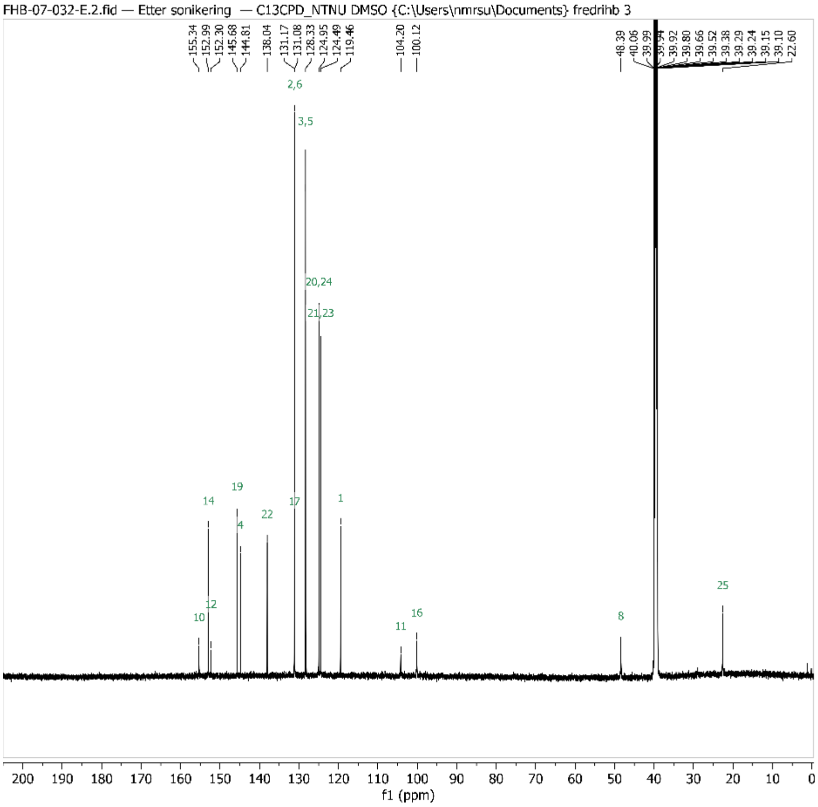

	Parameter	Value
1	Origin	Bruker BioSpin GmbH
2	Owner	nmrsu
3	Instrument	spect
4	Solvent	DMSO
5	Temperature	300.0
6	Pulse Sequence	zgpg30
7	Experiment	1D
8	Probe	Z117768_0061 (CP TCI 600S3 H-C/ N-D-05 Z)
9	Number of Scans	2048
10	Receiver Gain	197.1
11	Relaxation Delay	2.0000
12	Pulse Width	11.4000
13	Presaturation Frequency	
14	Acquisition Time	0.9088
15	Acquisition Date	2021-08-13T11:00:22
16	Modification Date	2021-08-13T11:00:22
17	Spectrometer Frequency	150.93
18	Spectral Width	36057.7
19	Lowest Frequency	-2938.0
20	Nucleus	13C
21	Acquired Size	32768
22	Spectral Size	65536
23	Digital Resolution	0.55

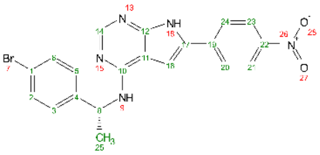

*Figure S26: ^{13}C NMR spectrum of compound **14** at 150 MHz in DMSO-d$_6$*

Compound 15

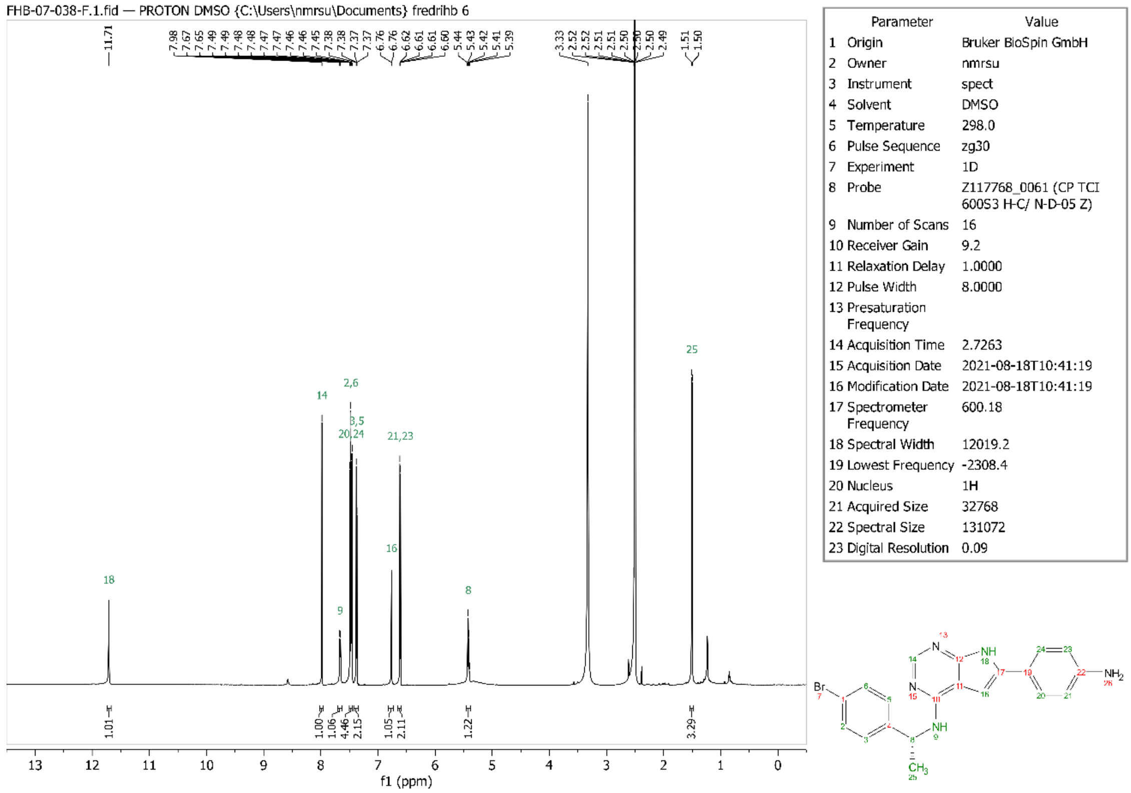

Parameter	Value
1 Origin	Bruker BioSpin GmbH
2 Owner	nmrsu
3 Instrument	spect
4 Solvent	DMSO
5 Temperature	298.0
6 Pulse Sequence	zg30
7 Experiment	1D
8 Probe	Z117768_0061 (CP TCI 600S3 H-C/ N-D-05 Z)
9 Number of Scans	16
10 Receiver Gain	9.2
11 Relaxation Delay	1.0000
12 Pulse Width	8.0000
13 Presaturation Frequency	
14 Acquisition Time	2.7263
15 Acquisition Date	2021-08-18T10:41:19
16 Modification Date	2021-08-18T10:41:19
17 Spectrometer Frequency	600.18
18 Spectral Width	12019.2
19 Lowest Frequency	-2308.4
20 Nucleus	1H
21 Acquired Size	32768
22 Spectral Size	131072
23 Digital Resolution	0.09

*Figure S27: ¹H NMR spectrum of compound **15** at 600 MHz in DMSO-d₆*

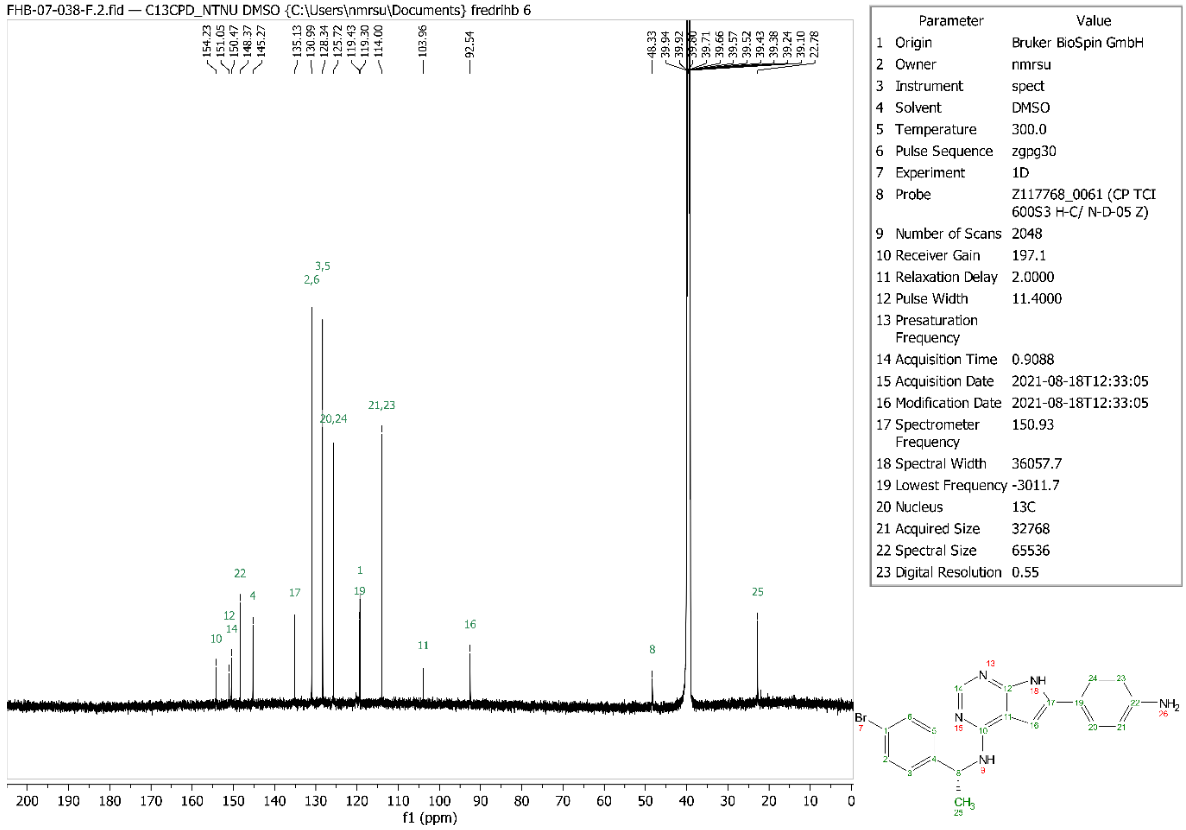

Parameter	Value
1 Origin	Bruker BioSpin GmbH
2 Owner	nmrsu
3 Instrument	spect
4 Solvent	DMSO
5 Temperature	300.0
6 Pulse Sequence	zgpg30
7 Experiment	1D
8 Probe	Z117768_0061 (CP TCI 600S3 H-C/ N-D-05 Z)
9 Number of Scans	2048
10 Receiver Gain	197.1
11 Relaxation Delay	2.0000
12 Pulse Width	11.4000
13 Presaturation Frequency	
14 Acquisition Time	0.9088
15 Acquisition Date	2021-08-18T12:33:05
16 Modification Date	2021-08-18T12:33:05
17 Spectrometer Frequency	150.93
18 Spectral Width	36057.7
19 Lowest Frequency	-3011.7
20 Nucleus	13C
21 Acquired Size	32768
22 Spectral Size	65536
23 Digital Resolution	0.55

*Figure S28: ¹³C NMR spectrum of compound **15** at 150 MHz in DMSO-d₆*

Compound 16

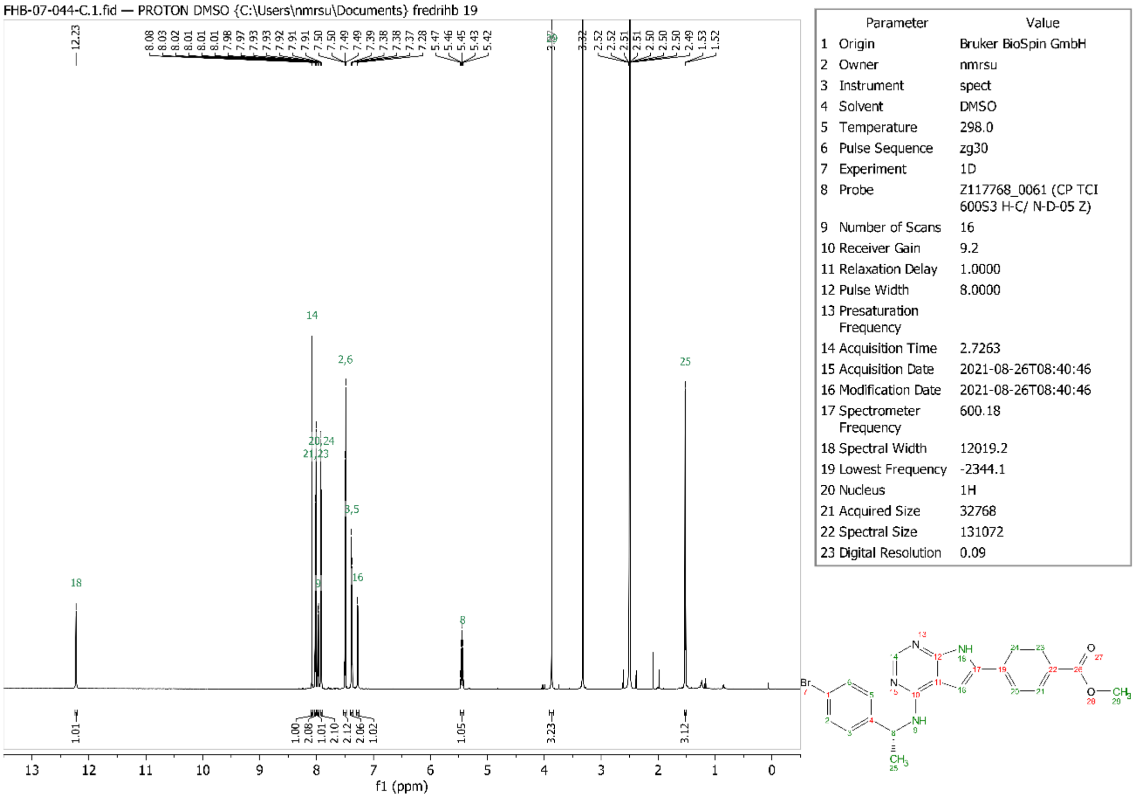

Figure S29: ¹H NMR spectrum of compound ***16*** *at 600 MHz in DMSO-d₆*

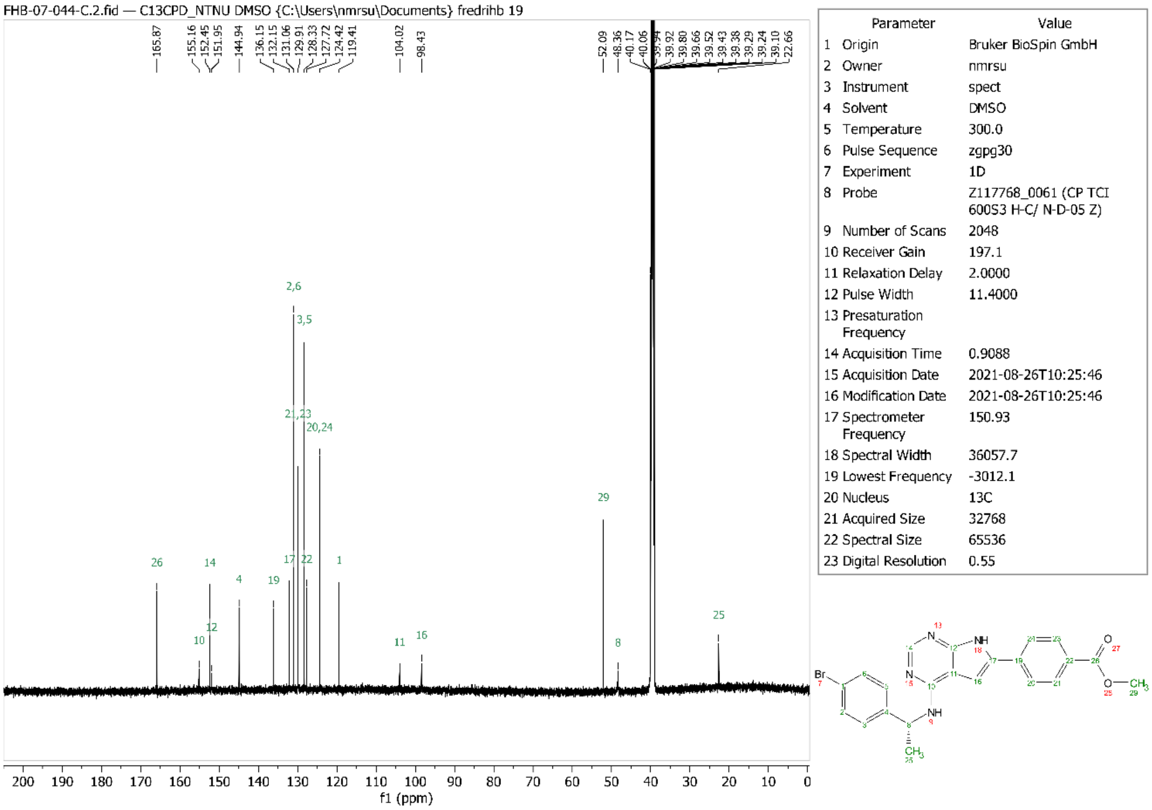

Figure S30: ¹³C NMR spectrum of compound ***16*** *at 150 MHz in DMSO-d₆*

Compound 17

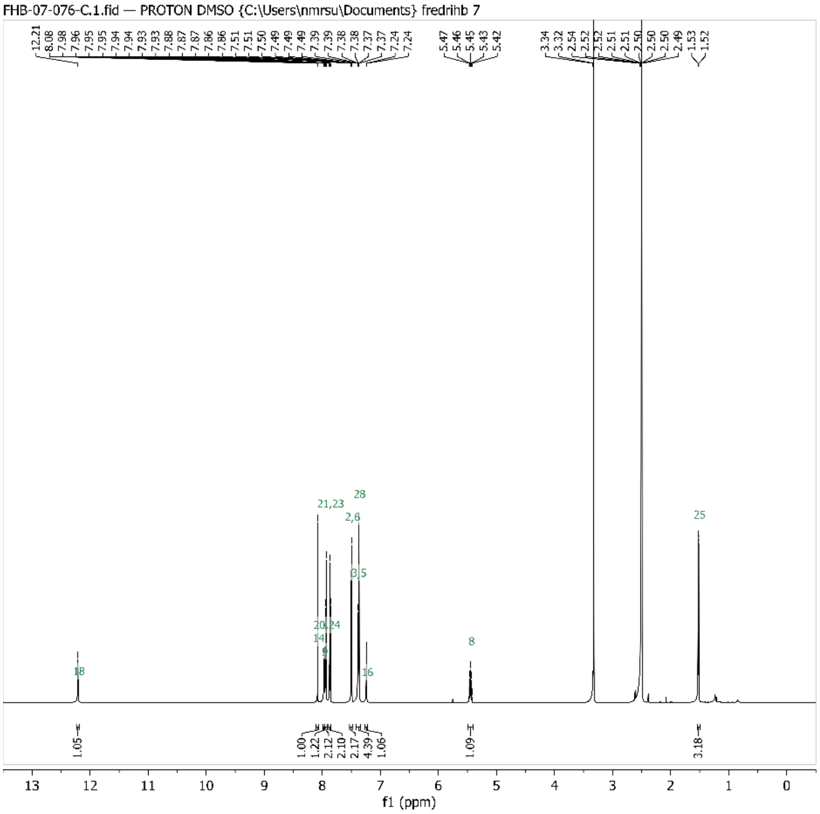

Parameter	Value
1 Origin	Bruker BioSpin GmbH
2 Owner	nmrsu
3 Instrument	spect
4 Solvent	DMSO
5 Temperature	298.0
6 Pulse Sequence	zg30
7 Experiment	1D
8 Probe	Z117768_0061 (CP TCI 600S3 H-C/ N-D-05 Z)
9 Number of Scans	16
10 Receiver Gain	9.2
11 Relaxation Delay	1.0000
12 Pulse Width	8.0000
13 Presaturation Frequency	
14 Acquisition Time	2.7263
15 Acquisition Date	2021-10-29T16:30:12
16 Modification Date	2021-10-29T16:30:12
17 Spectrometer Frequency	600.23
18 Spectral Width	12019.2
19 Lowest Frequency	-2303.2
20 Nucleus	1H
21 Acquired Size	32768
22 Spectral Size	131072
23 Digital Resolution	0.09

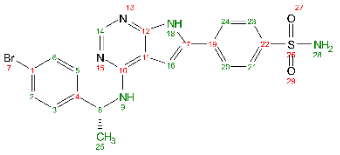

*Figure S31: ^{1}H NMR spectrum of compound **17** at 600 MHz in DMSO-d_6*

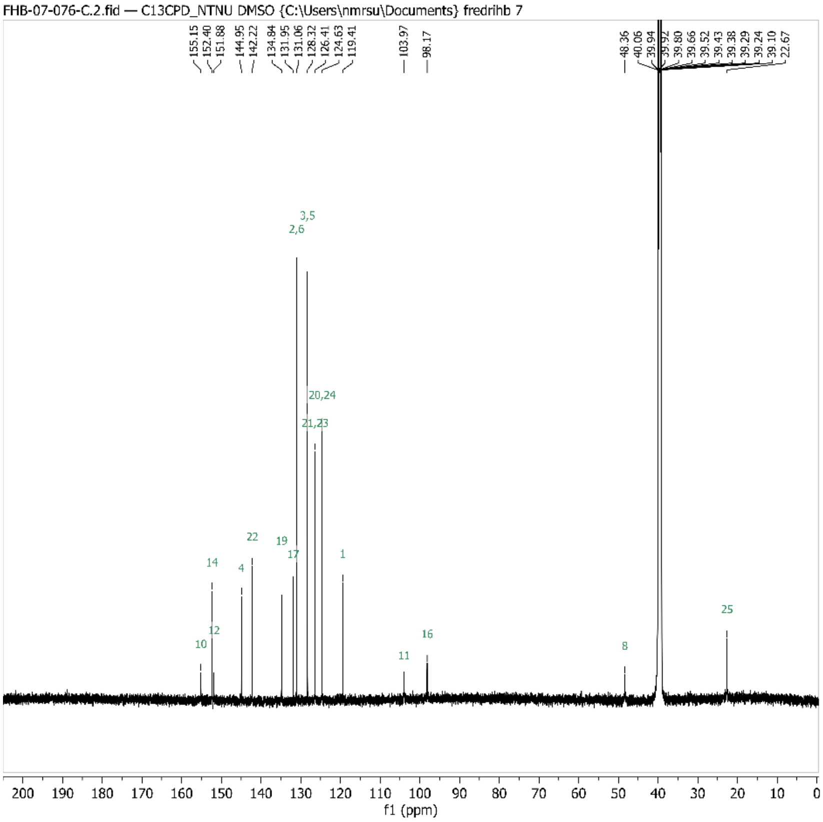

Parameter	Value
1 Origin	Bruker BioSpin GmbH
2 Owner	nmrsu
3 Instrument	spect
4 Solvent	DMSO
5 Temperature	300.0
6 Pulse Sequence	zgpg30
7 Experiment	1D
8 Probe	Z117768_0061 (CP TCI 600S3 H-C/ N-D-05 Z)
9 Number of Scans	2048
10 Receiver Gain	197.1
11 Relaxation Delay	2.0000
12 Pulse Width	11.4000
13 Presaturation Frequency	
14 Acquisition Time	0.9088
15 Acquisition Date	2021-10-29T18:14:07
16 Modification Date	2021-10-29T18:14:07
17 Spectrometer Frequency	150.94
18 Spectral Width	36057.7
19 Lowest Frequency	-2936.8
20 Nucleus	13C
21 Acquired Size	32768
22 Spectral Size	65536
23 Digital Resolution	0.55

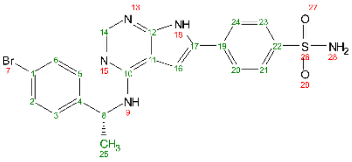

*Figure S32: ^{13}C NMR spectrum of compound **17** at 150 MHz in DMSO-d_6*

Compound 18

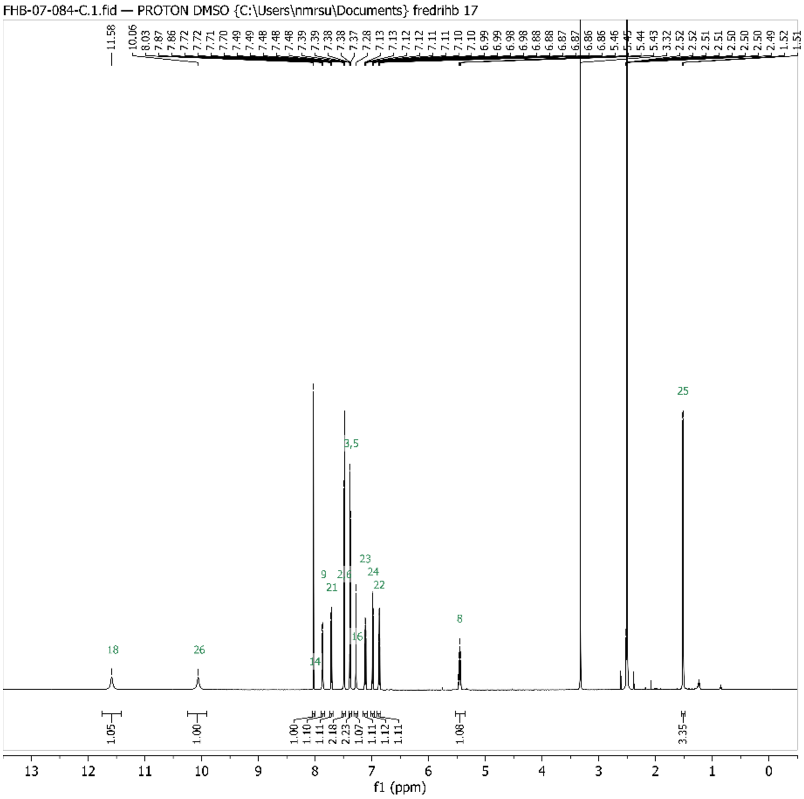

	Parameter	Value
1	Origin	Bruker BioSpin GmbH
2	Owner	nmrsu
3	Instrument	spect
4	Solvent	DMSO
5	Temperature	298.0
6	Pulse Sequence	zg30
7	Experiment	1D
8	Probe	Z117768_0061 (CP TCI 600S3 H-C/ N-D-05 Z)
9	Number of Scans	16
10	Receiver Gain	9.2
11	Relaxation Delay	1.0000
12	Pulse Width	8.0000
13	Presaturation Frequency	
14	Acquisition Time	2.7263
15	Acquisition Date	2021-11-03T13:03:13
16	Modification Date	2021-11-03T13:03:13
17	Spectrometer Frequency	600.23
18	Spectral Width	12019.2
19	Lowest Frequency	-2307.9
20	Nucleus	1H
21	Acquired Size	32768
22	Spectral Size	131072
23	Digital Resolution	0.09

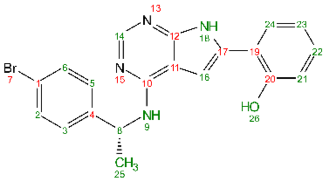

*Figure S33: ¹H NMR spectrum of compound **18** at 600 MHz in DMSO-d₆*

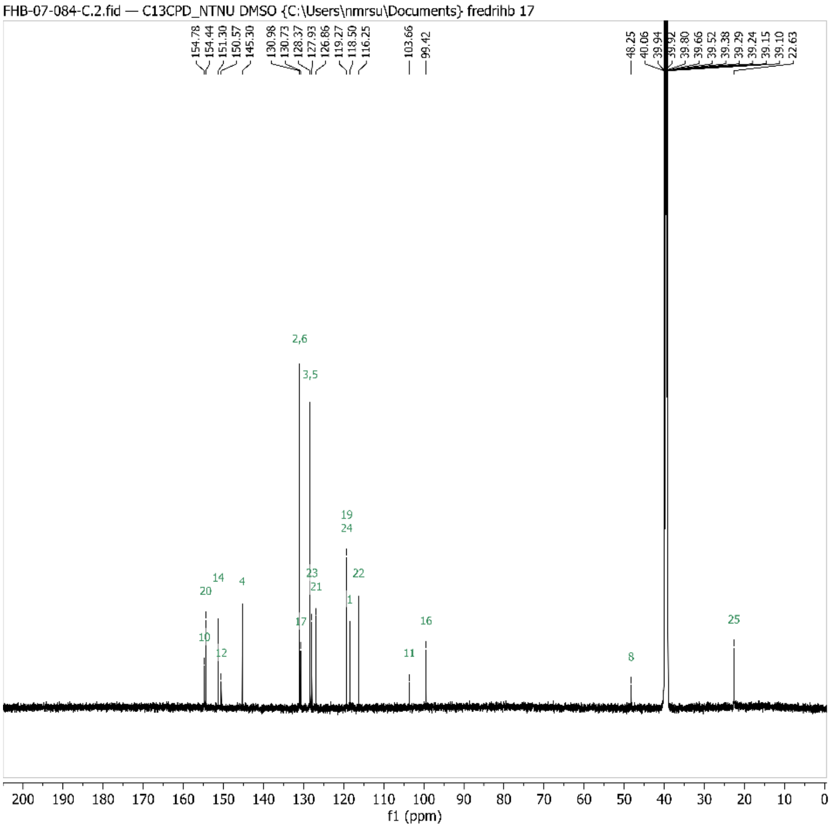

	Parameter	Value
1	Origin	Bruker BioSpin GmbH
2	Owner	nmrsu
3	Instrument	spect
4	Solvent	DMSO
5	Temperature	300.0
6	Pulse Sequence	zgpg30
7	Experiment	1D
8	Probe	Z117768_0061 (CP TCI 600S3 H-C/ N-D-05 Z)
9	Number of Scans	2048
10	Receiver Gain	197.1
11	Relaxation Delay	2.0000
12	Pulse Width	11.4000
13	Presaturation Frequency	
14	Acquisition Time	0.9088
15	Acquisition Date	2021-11-03T14:47:05
16	Modification Date	2021-11-03T14:47:05
17	Spectrometer Frequency	150.94
18	Spectral Width	36057.7
19	Lowest Frequency	-3010.9
20	Nucleus	13C
21	Acquired Size	32768
22	Spectral Size	65536
23	Digital Resolution	0.55

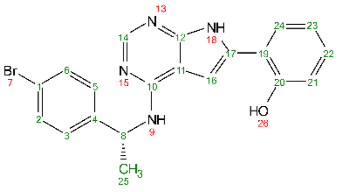

*Figure S34: ¹³C NMR spectrum of compound **18** at 150 MHz in DMSO-d₆*

Compound 19

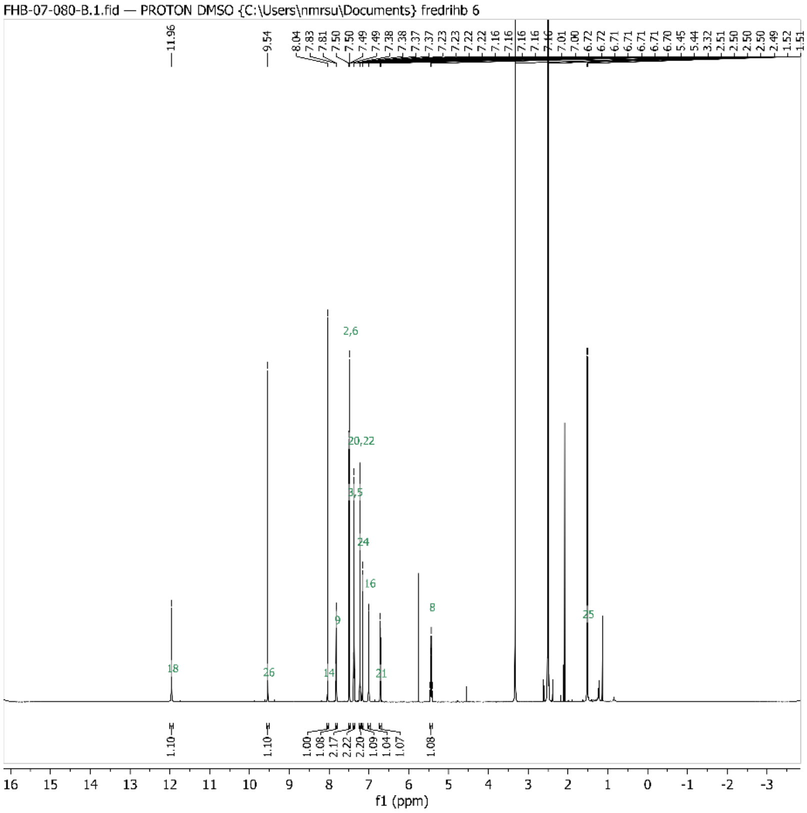

Parameter		Value
1	Title	FHB-07-080-B.1.fid
2	Origin	Bruker BioSpin GmbH
3	Owner	nmrsu
4	Instrument	spect
5	Solvent	DMSO
6	Temperature	298.0
7	Pulse Sequence	zg30
8	Experiment	1D
9	Probe	Z117768_0061 (CP TCI 600S3 H-C/ N-D-05 Z)
10	Number of Scans	16
11	Receiver Gain	9.2
12	Relaxation Delay	1.0000
13	Pulse Width	8.0000
14	Presaturation Frequency	
15	Acquisition Time	2.7263
16	Acquisition Date	2021-10-29T12:55:28
17	Modification Date	2021-10-29T12:55:28
18	Spectrometer Frequency	600.23
19	Spectral Width	12019.2
20	Lowest Frequency	-2307.9
21	Nucleus	1H
22	Acquired Size	32768
23	Spectral Size	131072
24	Digital Resolution	0.09

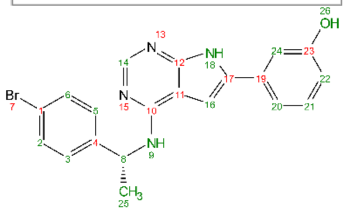

*Figure S35: ^{1}H NMR spectrum of compound **19** at 600 MHz in DMSO-d6*

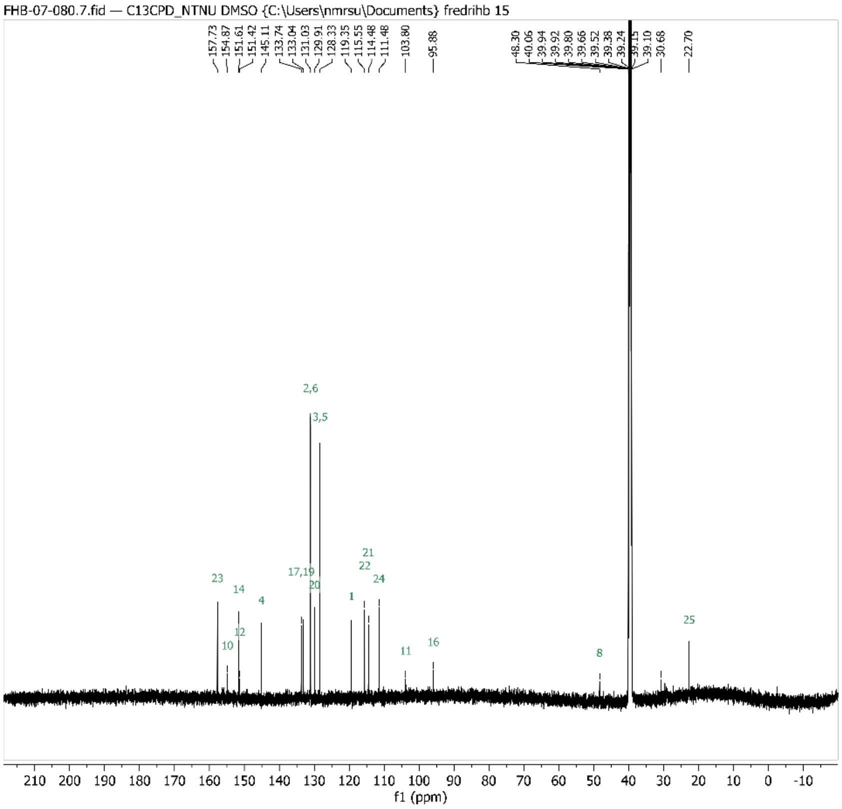

Parameter		Value
1	Title	FHB-07-080.7.fid
2	Origin	Bruker BioSpin GmbH
3	Owner	nmrsu
4	Instrument	spect
5	Solvent	DMSO
6	Temperature	300.0
7	Pulse Sequence	zgpg30
8	Experiment	1D
9	Probe	Z117768_0061 (CP TCI 600S3 H-C/ N-D-05 Z)
10	Number of Scans	8192
11	Receiver Gain	197.1
12	Relaxation Delay	2.0000
13	Pulse Width	11.4000
14	Presaturation Frequency	
15	Acquisition Time	0.9088
16	Acquisition Date	2021-11-05T20:22:32
17	Modification Date	2021-11-05T20:22:32
18	Spectrometer Frequency	150.94
19	Spectral Width	36057.7
20	Lowest Frequency	-2936.8
21	Nucleus	13C
22	Acquired Size	32768
23	Spectral Size	65536
24	Digital Resolution	0.55

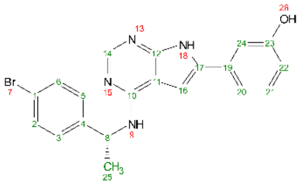

*Figure S36: ^{13}C NMR spectrum of compound **19** at 150 MHz in DMSO-d6*

Compound 20

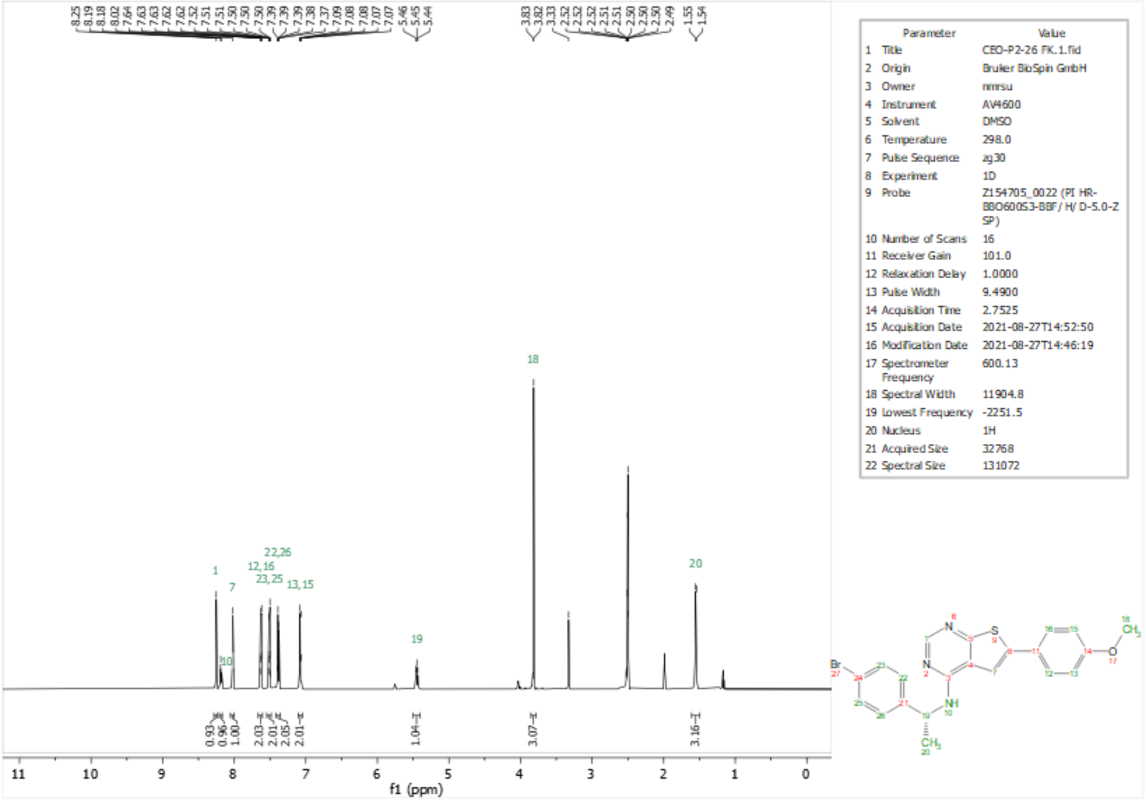

Figure S37: ^{1}H NMR spectrum of compound ***20*** *at 600 MHz in DMSO-d_6*

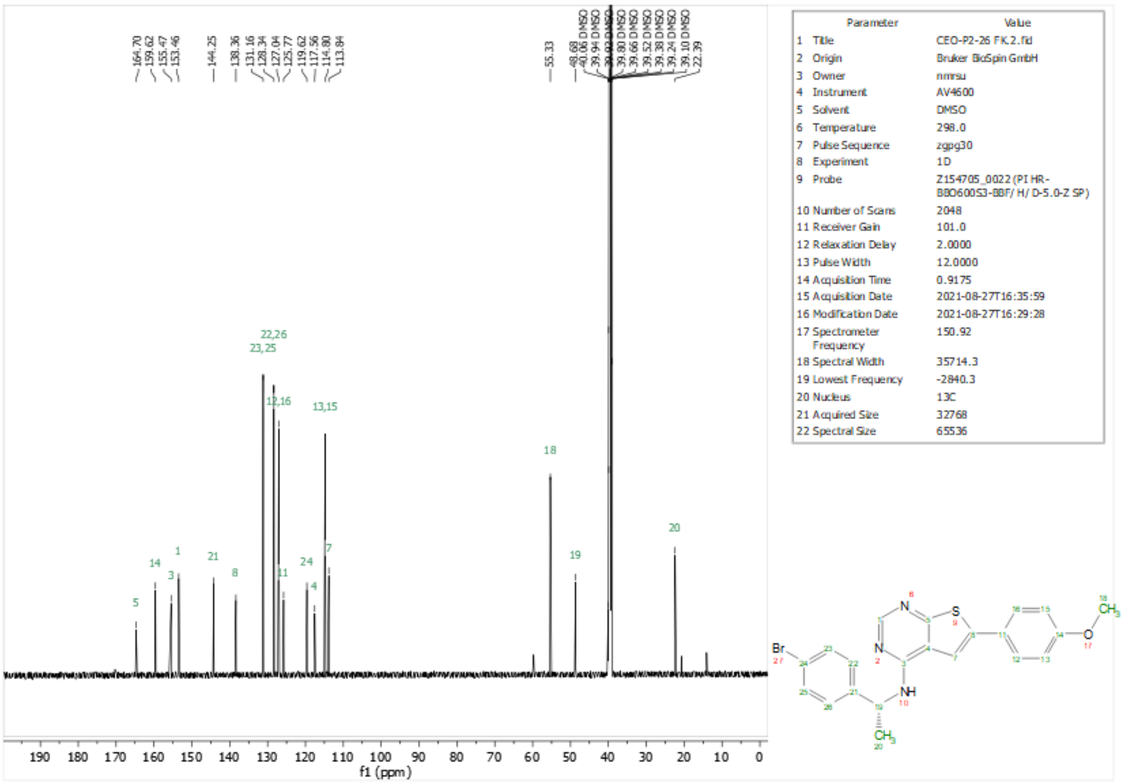

Figure S38: ^{13}C NMR spectrum of compound ***20*** *at 150 MHz in DMSO-d_6*

Compound 21

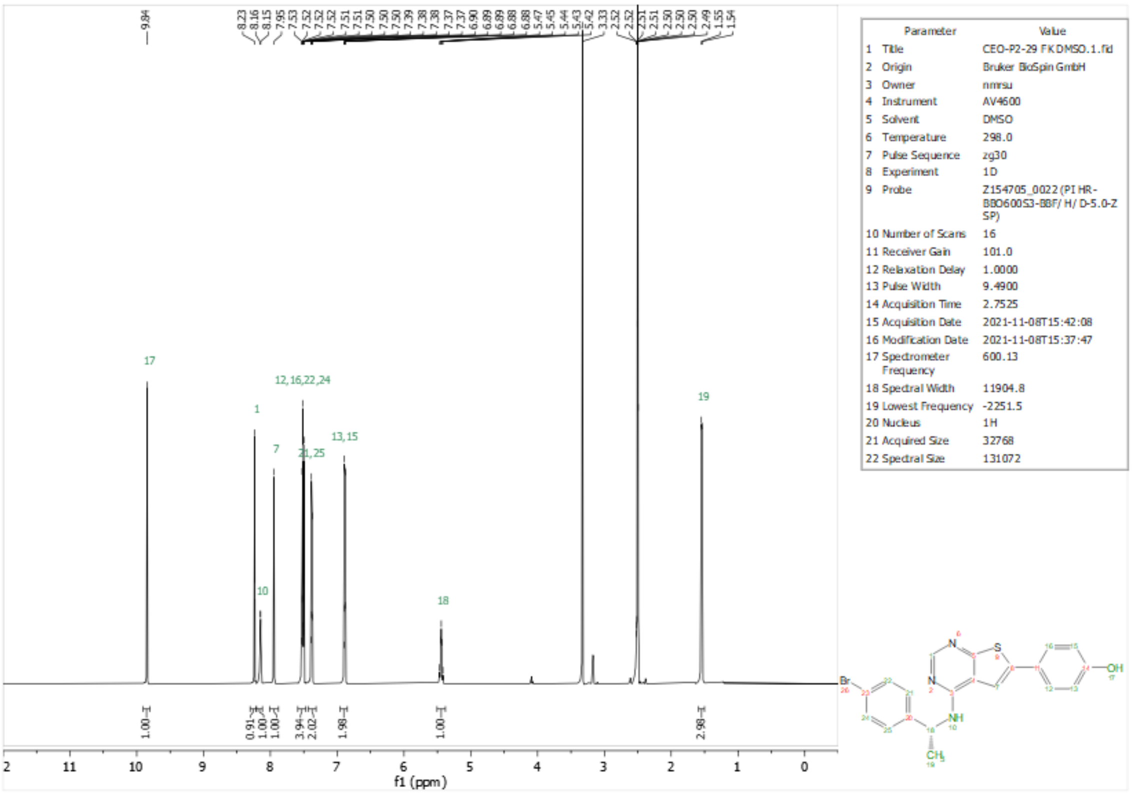

*Figure S39: ^{1}H NMR spectrum of compound **21** at 600 MHz in DMSO-d6*

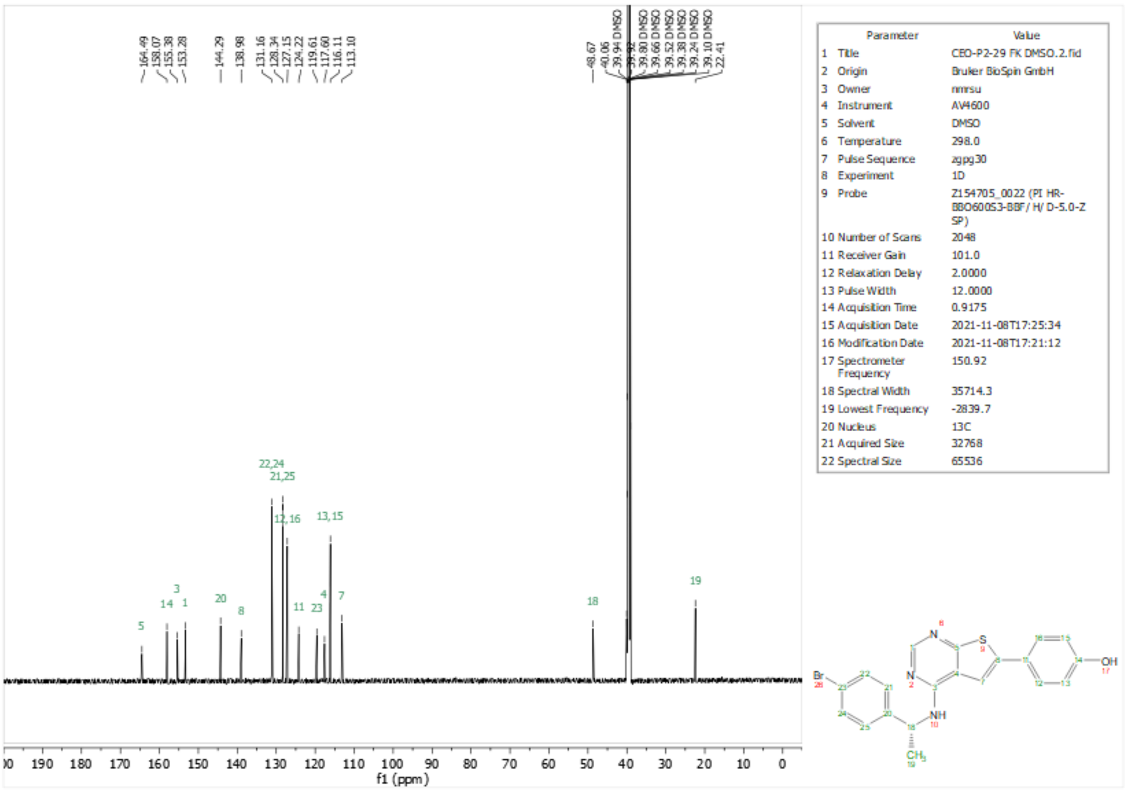

*Figure S40: ^{13}C NMR spectrum of compound **21** at 150 MHz in DMSO-d6.*

Comparison of TMPK folding

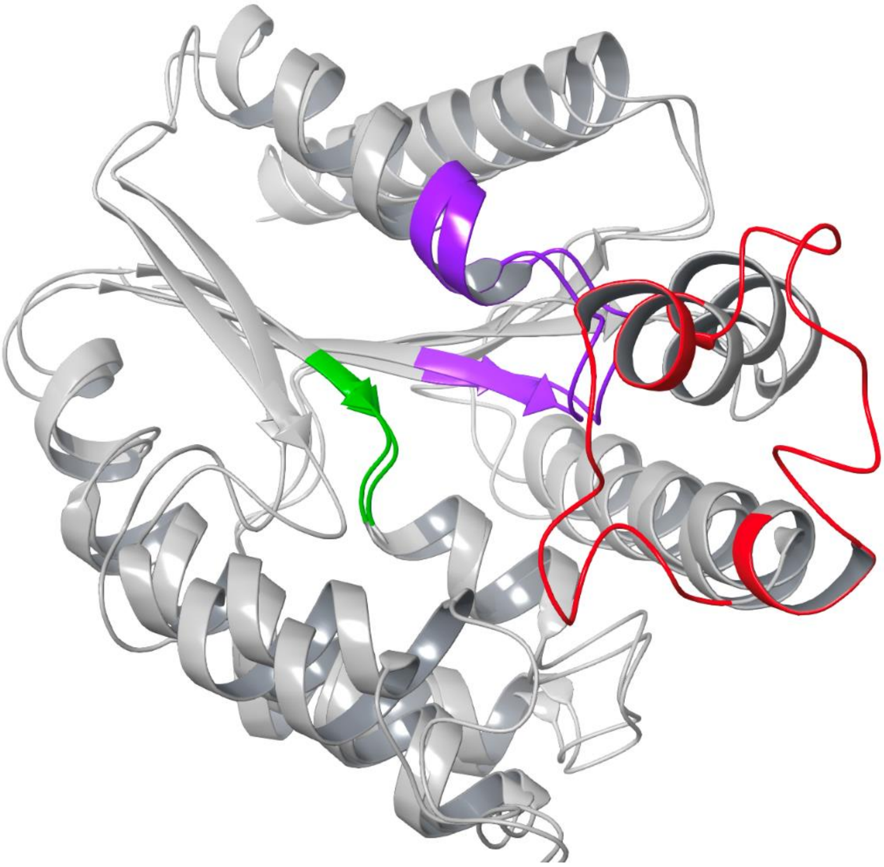

Figure S41. The crystal structures of TMPK from E. coli (4TMK.pdb) and S. aureus (4GSY.pdb) overlayed with the P-loops hightailed in purple, the DRX motif highlighted in green and the lid-structures highlighted in red. (Made using the Schrodinger Maestro suite (version 13.1.144, Release 2022-1)

References

1. Liang, X.; Huang, Y.; Zang, J.; Gao, Q.; Wang, B.; Xu, W.; Zhang, Y., Design, synthesis and preliminary biological evaluation of 4-aminopyrazole derivatives as novel and potent JAKs inhibitors. *Bioorganic & Medicinal Chemistry* **2016,** *24* (12), 2660-2672.
2. Reiersølmoen, A. C.; Han, J.; Sundby, E.; Hoff, B. H., Identification of fused pyrimidines as interleukin 17 secretion inhibitors. *Eur. J. Med. Chem.* **2018,** *155*, 562-578.
3. Blindheim, F. H.; Malme, A. T.; Dalhus, B.; Sundby, E.; Hoff, B. H., Synthesis and Evaluation of Fused Pyrimidines as E. coli Thymidylate Monophosphate Kinase Inhibitors. *ChemistrySelect* **2021,** *6* (45), 12852-12857.
4. Klečka, M.; Poštová Slavětínská, L.; Hocek, M., Modification of Pyrrolo[2,3-d]pyrimidines by C–H Borylation Followed by Cross-Coupling or Other Transformations: Synthesis of 6,8-Disubstituted 7-Deazapurine Bases. *Eur. J. Org. Chem.* **2015,** *2015* (36), 7943-7961.
5. Bugge, S.; Kaspersen, S. J.; Sundby, E.; Hoff, B. H., Route selection in the synthesis of C-4 and C-6 substituted thienopyrimidines. *Tetrahedron* **2012,** *68* (45), 9226-9233.
6. Bugge, S.; Skjønsfjell, E. M.; Willumsen, F. B.; Sundby, E.; Hoff, B. H., Improved and Scalable Preparation of 6-Bromo-4-Chlorothieno[2,3-d]Pyrimidine. *Chem. Heterocycl. Compd.* **2014,** *50* (8), 1177-1187.
